# Optimizing copper nanoparticles with a carbon shell for enhanced electrochemical CO_2_ reduction to ethanol†

**DOI:** 10.1039/d3sc04061e

**Published:** 2023-11-24

**Authors:** Ting Yao, Wei Xia, Shitao Han, Shuaiqiang Jia, Xue Dong, Min Wang, Jiapeng Jiao, Dawei Zhou, Jiahao Yang, Xueqing Xing, Chunjun Chen, Mingyuan He, Haihong Wu, Buxing Han

**Affiliations:** a Shanghai Key Laboratory of Green Chemistry and Chemical Processes, School of Chemistry and Molecular Engineering, East China Normal University Shanghai 200062 China wxia@chem.ecnu.edu.cn hhwu@chem.ecnu.edu.cn hanbx@iccas.ac.cn; b Institute of Eco-Chongming 20 Cuiniao Road, Chenjia Town, Chongming District Shanghai 202162 China hhwu@chem.ecnu.edu.cn; c Beijing National Laboratory for Molecular Sciences, CAS Key Laboratory of Colloid and Interface and Thermodynamics, CAS Research/Education Center for Excellence in Molecular Sciences, Institute of Chemistry, Chinese Academy of Sciences Beijing 100190 China hanbx@iccas.ac.cn; d Beijing Synchrotron Radiation Facility, Institute of High Energy Physics, Chinese Academy of Sciences Beijing Municipality 100049 China

## Abstract

The electrochemical reduction of carbon dioxide (CO_2_RR) holds great promise for sustainable energy utilization and combating global warming. However, progress has been impeded by challenges in developing stable electrocatalysts that can steer the reaction toward specific products. This study proposes a carbon shell coating protection strategy by an efficient and straightforward approach to prevent electrocatalyst reconstruction during the CO_2_RR. Utilizing a copper-based metal–organic framework as the precursor for the carbon shell, we synthesized carbon shell-coated electrocatalysts, denoted as Cu-*x-y*, through calcination in an N_2_ atmosphere (where *x* and *y* represent different calcination temperatures and atmospheres: N_2_, H_2_, and NH_3_). It was found that the faradaic efficiency of ethanol over the catalysts with a carbon shell could reach ∼67.8%. In addition, the catalyst could be stably used for more than 16 h, surpassing the performance of Cu-600-H_2_ and Cu-600-NH_3_. Control experiments and theoretical calculations revealed that the carbon shell and Cu–C bonds played a pivotal role in stabilizing the catalyst, tuning the electron environment around Cu atoms, and promoting the formation and coupling process of CO*, ultimately favoring the reaction pathway leading to ethanol formation. This carbon shell coating strategy is valuable for developing highly efficient and selective electrocatalysts for the CO_2_RR.

## Abstract 2

Employing a carbon shell-coating structure and Cu–C bonds enhanced the stability of the catalyst, promoted the formation and coupling process of CO*, ultimately favoring the reaction pathway to ethanol formation.

## Graphical abstract

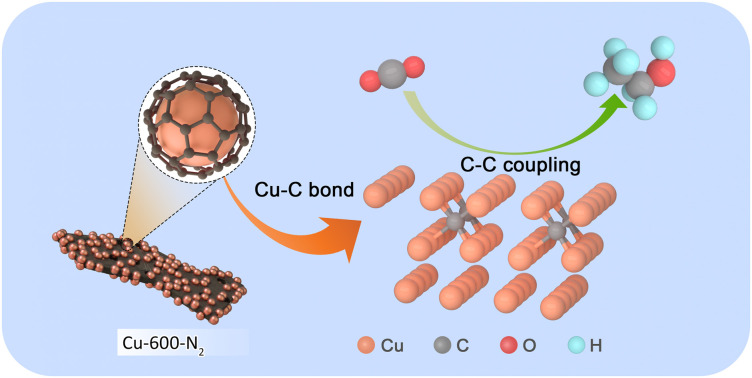

## Introduction

Electrocatalytic carbon dioxide reduction (CO_2_RR), powered by clean and renewable energy resources, is a widely explored reaction due to its promise as a sustainable approach to mitigating global warming and promoting sustainable energy utilization.^1–3^ Among the various CO_2_RR products, the production of high-value multicarbon (C_2+_) compounds has garnered significant attention from researchers. However, most of these technologies still require better catalysts to support electrochemical reactions. In particular, there is a lack of in-depth understanding of a catalyst's dynamic structure under reaction conditions, which is however critically needed to inform rational catalyst design.^4–6^

Copper (Cu) has been extensively studied in the CO_2_RR due to its ability to induce CO* dimerization (where * represents the adsorption state), especially towards deep-reduction products (*e.g.*, eight-electron transfer for methane (CH_4_) and twelve-electron transfer for ethylene (C_2_H_4_) and ethanol (C_2_H_5_OH)).^7–9^ Various methods, such as modifications of the microstructure,^10,11^ hydrophobicity,^12^ defects,^13^ facets,^14–16^ and heteroatom doping,^17,18^ have been applied to enhance C_2+_ product selectivity and catalytic activity. However, there is also increasing evidence that significant catalyst restructuring can already occur as soon as the Cu-based catalysts are placed into an electrolyte, and these changes impact the subsequent catalyst activity and selectivity during the reaction.^47^ Despite the increasing utilization of heteroatom-induced modification of copper catalysts as an electrode preparation method to enhance catalyst stability, there have been limited investigations into the detailed analysis of morphological transformations and the exploration of strategies for controlled synthesis of the most advantageous nanostructures for a specific electrocatalytic process.

Carbon shells serve as effective physical barriers between nanocatalysts to address catalyst restructuring, reducing particle aggregation, and preventing oxidation. In the past decade, carbon-shell-encapsulated nanoparticles have shown great potential as electrocatalysts for electrochemical energy applications. For example, in the Pt–aniline complex, carbon shells are simply encapsulated on Pt nanoparticles to enhance the long-term stability of proton exchange membrane fuel cells,^19^ and carbon-encapsulated ordered PtFe nanoparticles exhibit exceptional activity and durability as electrocatalysts for fuel cell applications.^20^ Additionally, several studies have indicated that the interface hybridization between carbon matrices and transition metals significantly influences the activity and selectivity of electrocatalysts.^20^ This is attributed to the modulation of the electronic structure of the external carbon shell by the core metal particles, which leads to significant changes in the electron density of the carbon shells.^21,22^

In this study, we present a novel carbon shell (C shell) coating protection strategy, employing an efficient and straightforward approach, aimed at preventing electrocatalyst reconstruction during the CO_2_RR. A copper-based metal–organic framework (Cu-MOF) was selected as the precursor for the C shell, and the carbon shell was realized through calcination in an N_2_ atmosphere. Furthermore, Cu-MOFs were also exposed to different gas atmospheres, such as H_2_ and NH_3_, at various temperatures to demonstrate the significance of the C shell. The resulting catalysts were labeled as Cu-*x-y*, where *x* and *y* represent the calcination temperature and atmosphere, respectively. It was found that Cu-600-N_2_ consisted of Cu nanoparticles with a 2 nm thick carbon shell and Cu–C bonds, and it showed excellent stability and efficiency for the CO_2_-to-ethanol reaction. The faradaic efficiency (FE) of ethanol could be as high as 67.8%, much better than that of Cu-600-H_2_ and Cu-600-NH_3_. The carbon shell and Cu–C bonds played a crucial role in stabilizing the catalyst, tuning the electron environment around Cu atoms, promoting the formation of CO*, and following C–C coupling to generate ethanol.

## Results and discussion

### Synthesis and characterization of C shell-protected Cu

**Fig. 1:**
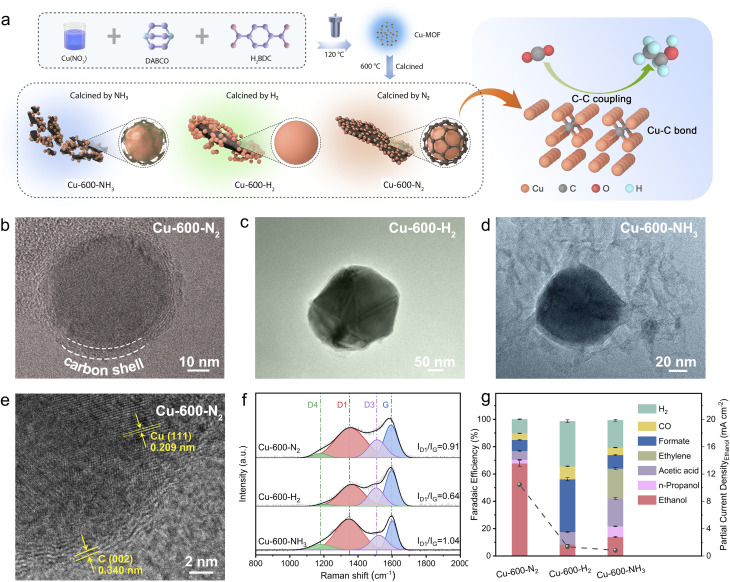
(a) The schematic illustration of the preparation of catalysts. TEM characterization of (b) Cu-600-N_2_ (c) Cu-600-H_2,_ and (d) Cu-600-NH_3_, respectively. (e) HR-TEM images of Cu-600-N_2_. (f) Raman spectra and (g) FE over Cu-600-N_2_, Cu-600-H_2,_ and Cu-600-NH_3_ in CO_2_-saturated 0.1 M KHCO_3_ aqueous solutions using an H-cell at −0.8 V (*vs.* RHE).

First, Cu-MOFs were synthesized using a solvothermal approach based on a bottom-up procedure, which followed a previously reported procedure with certain modifications.^48,49^ Terephthalic acid (H_2_BDC), 1,4-diazabicyclooctane (DABCO), and copper nitrate Cu(NO_3_)_2_ were utilized for the synthesis of the Cu-MOFs, as illustrated in Fig. 1a. Each Cu^2+^ cation in the Cu-MOFs is coordinated with four oxygen atoms from H_2_BDC and one nitrogen atom from DABCO molecules within a single building unit. The resulting Cu-MOFs had a size of approximately 2 μm (Fig. S1†), and nitrogen adsorption experiments reveal a Brunauer–Emmett–Teller (BET) surface area of 223.5967 m^2^ g^−1^ (Fig. S2†). The X-ray diffraction (XRD) pattern displayed a well-crystallized structure of Cu-MOFs, consistent with the previous results (Fig. S3†).^48,49^ To produce the Cu-*x*-N_2_ catalysts, the precursor Cu-MOFs were pyrolyzed at various temperatures (400, 500, 600, 700, 800 and 900 °C) in an N_2_ environment. Using the same approach, reference compounds Cu-*x*-H_2_ and Cu-*x*-NH_3_ were also produced under H_2_ and NH_3_ atmospheres, respectively. As shown in Fig. S4a,† the XRD patterns of all three catalysts Cu-600-N_2_, Cu-600-H_2,_ and Cu-600-NH_3_ displayed three distinct diffraction peaks at approximately 43.3°, 50.5°, and 74.1°, which correspond to Cu (111), Cu (200), and Cu (220) faces of metallic Cu (JCPDS no. 04-0836), respectively. The results of inductively coupled plasma (ICP) analysis are presented in Table S1,† which indicates that there was hardly any difference in copper loading between the three catalysts.

The TEM and SEM images showed that Cu^2+^ was converted into monodispersed copper nanospheres with a polycrystalline structure after heat treatment (Fig. 1b–d and S4b–d†). The morphology of the three catalysts calcined in different atmospheres differed significantly. First, as can be seen from Fig. 1b–d and S4b–d,† Cu-600-N_2,_ and Cu-600-NH_3_ were composed of nanoparticles with an average size of less than 100 nm, while Cu-600-H_2_ was composed of larger-sized sintered particles (Fig. S5–S7†). TEM characterization (Fig. S5†) indicates that the particle size of Cu nanoparticles (Cu NPs) deposited on carbon ranged from 20 to 50 nm in the Cu-600-N_2_ catalyst. In addition, the combination of carbon and Cu-NPs depended on atmospheric conditions in the calcination process. As shown in Fig. 1b and e, the interplanar spacings of the carbon layers and the Cu NPs were 0.34 nm and 0.210 nm, respectively, consistent with the C (002) and Cu (111) crystal planes.^20–22^ The Cu NPs, covered with an about 3 nm-thick carbon shell, were seamlessly incorporated into the C framework in Cu-600-N_2_. Previous research has demonstrated that thin carbon shells possess permeability for small molecules and do not impede the mass transport of reactants.^23–25^ The elemental mapping also indicated the tight binding of Cu and carbon in Cu-600-N_2_ (Fig. S9–S11†). In contrast, due to the reducibility of H_2_, the carbon is squeezed out of the Cu-MOF precursor, resulting in the apparent separation of carbon and Cu-NPs in Cu-600-H_2_ (Fig. 1c). Additionally, in Cu-600-NH_3_ (Fig. 1d), NH_3_ inhibited the sintering of carbon particles, resulting in an incomplete and fragmented carbon layer.^26^

Raman spectroscopy was used to determine the reason for the discrepancy. As shown in Fig. 1f, the two broad peaks at 1348.2 and 1596.1 cm^−1^ correspond to the D and G bands, respectively. The D band refers to sp^2^ hybridized carbon and material defects, while the G band is related to an ideal graphitic lattice. Therefore, the intensity ratio between the D and G bands (*I*_D_/*I*_G_) can be used to determine the degree of graphitization of carbon materials.^23,24^ The smaller the ratio is, the higher the degree of graphitization. By comparing the intensity ratios of several Raman bands, we found that the *I*_D_/*I*_G_ ratios increased from Cu-600-H_2_ (0.64) to Cu-600-N_2_ (0.91) and Cu-600-NH_3_ (1.04). This suggests that Cu-600-NH_3_ had a more disordered carbon support, and Cu-600-H_2_ had the most significant graphitization degree. Thus, different atmospheres resulted in various states of carbon layers, with ordered organization resulting in carbon separation, and disordered carbon may result in diminished conductivity and confinement ability.

The specific surface area and the pore-size distribution of the catalysts are further investigated using the N_2_adsorption–desorption isotherm at 77 K which is shown in Fig. S12a.† The isotherms are classified as the type IV category, displaying an H3 hysteresis loop, which indicates a mesoporous structure.^50^ The pore size distribution estimated by the nonlocal density functional theory method shows that the primary pore sizes are 4–6 nm for Cu-600-N_2_, 1.5 nm for Cu-600-NH_3,_ and 1.6 nm for Cu-600-H_2_ (Fig. S12b†). Furthermore, mesoporous Cu-600-N_2_ exhibits a notably high BET surface area of 1004.63 m^2^ g^−1^ and a sufficient pore volume of 2.96 cm^3^ g^−1^ (Table S2†). This extensive surface area offers a greater number of active sites and reactant transmission channels. To further evaluate the adsorption strength of CO_2_ on the catalyst surface, CO_2_ temperature-programmed desorption (CO_2_ TPD) has been conducted under identical mass loading conditions (Fig. S13a†). The results indicate that the desorption temperature and peak intensity of chemically adsorbed CO_2_ on Cu-600-N_2_ are higher than those of Cu-600-H_2_ and Cu-600-NH_3_, indicating stronger adsorption capability of Cu-600-N_2_ for CO_2_.^51^ Furthermore, the result of CO_2_ adsorption isotherms in Fig. S13b† is corroborated with the CO_2_ TPD analyses.

Control experiments were conducted to investigate the initial impact of different carbon layer states on the CO_2_RR, and the results are presented in Fig. 1g. Compared to samples Cu-600-H_2_ and Cu-600-NH_3_, Cu-600-N_2_ consisting of Cu nanoparticles encapsulated in a thin and complete carbon shell, exhibited significantly enhanced ethanol selectivity. This improvement is possibly attributed to the synergistic effect between Cu nanoparticles and the carbon shell in Cu-600-N_2_, and a detailed analysis will be provided below.

### Structural characterization

**Fig. 2:**
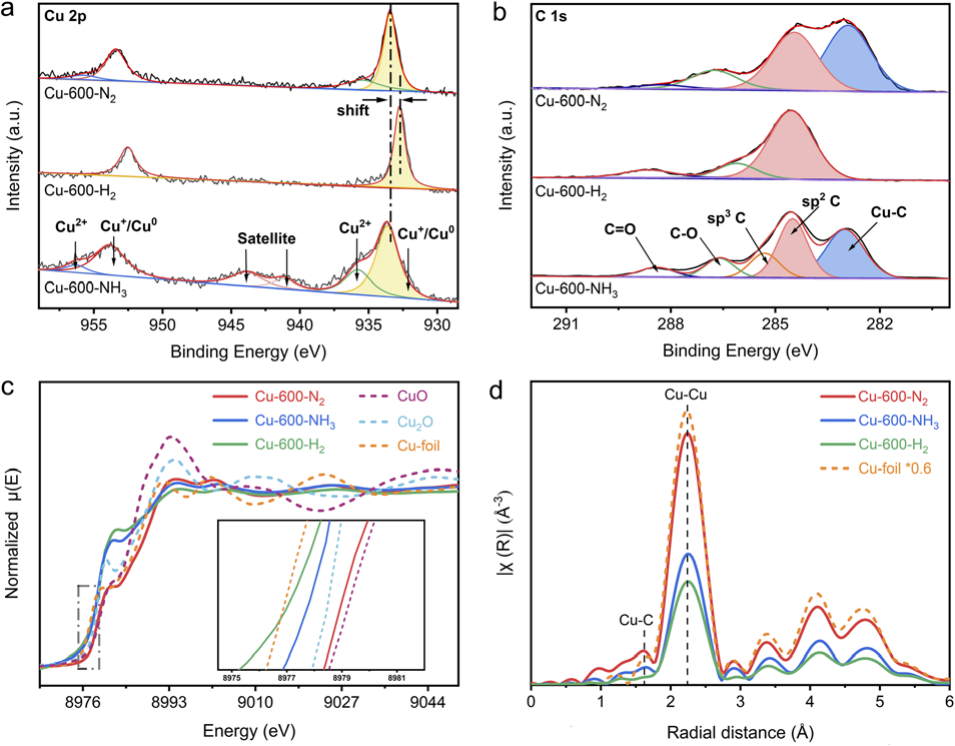
(a) High-resolution Cu 2p XPS spectra and (b) C 1s XPS spectra of Cu-600-NH_3_, Cu-600-N_2_, and Cu-600-H_2_. (c) Normalized Cu K-edge XANES spectra and (d) Cu K-edge FT-EXAFS spectra in the *R*-space of Cu-600-NH_3_, Cu-600-N_2_, Cu-600-H_2_, Cu foil, CuO, and Cu_2_O.

To investigate the impact of various calcination atmospheres on the performance of the catalysts, a series of Cu-based catalysts pyrolyzed under different atmospheres were characterized as follows. First, it is evident from the results discussed above that different atmospheres resulted in varying degrees of graphitization, with the degree of graphitization being Cu-600-H_2_ > Cu-600-N_2_ > Cu-600-NH_3_. In the high-resolution Cu 2p X-ray photoelectron spectra (XPS) (Fig. 2a), the peaks with a binding energy (BE) of around 952.2 and 932.3 eV are assigned to Cu(i)/Cu(0) species, whereas the peaks at 954.3 and 934.5 eV should be attributed to Cu(ii).^25,26^ Cu-600-N_2_ and Cu-600-NH_3_ exhibited shifted metallic Cu(0) peaks compared with Cu-600-H_2_, indicating a higher valence state of Cu in the former. This also signifies that the interfacial charge may be transferred from Cu nanoparticles to the carbon support.^27,28^ Based on the relative intensities of the C 1s peaks in Fig. 2b, the C 1s XPS spectrum was fitted with six peaks. The deconvoluted C 1s peaks at 283.2, 284.6, 285.5, 286.8, and 288.5 correspond to Cu–C, sp^2^–C, sp^3^–C, C–O, and C=O groups,^29–31^ respectively. Notably, the defective sp^3^ carbon atoms were introduced into Cu-600-NH_3_. This coincides with the Raman and SEM results that Cu-600-NH_3_ had the least graphitized carbon support. The percentage of sp^2^ and sp^3^-hybridized C atoms was estimated as shown in Table S3.† Moreover, due to charge transfer at the interface, a typical “shoulder” appears at the low binding energy of C 1s. A spilled peak at 283.2 in Cu-600-N_2_ and Cu-600-NH_3_ indicates a strong interaction between metal and carbide (M–C bond).^29^ Additionally, the Cu–C levels caused by N_2_ are about one-fold larger than those induced by NH_3_. Our research revealed that the nitrogen environment might create a certain content of carbon defects and enhance the synergy between the M–C bonds. The effect of other gases on the properties of the catalysts was also investigated. NH_3_ hindered the sintering of carbon particles, resulting in the carbon support with poor graphitization and electroconductivity. On the other hand, in an H_2_ environment, Cu-MOF precursors tend to produce a high degree of graphitization.^26,27^

To further examine the electronic structures and chemical configurations of catalysts Cu-600-N_2_, Cu-600-H_2_ and Cu-600-NH_3_, X-ray absorption near-edge structure (XANES) and extended X-ray absorption fine structure (EXAFS) spectroscopies were performed. Fig. 2c shows the Cu K-edge XANES spectra of Cu-600-N_2_, Cu-600-H_2_ and Cu-600-NH_3_, alongside CuO, Cu_2_O, and Cu foil references. Compared to Cu-600-H_2_ and Cu-600-NH_3_, the absorption edge position of Cu-600-N_2_ is positively shifted and virtually identical to that of CuO, suggesting a higher oxidation state of the copper species and a tendency toward a +2 valence state. This is consistent with the XPS results. In addition, the edge positions of the spectra acquired on Cu-600-H_2_ and Cu-600-NH_3_ are between Cu_2_O and Cu, suggesting that the oxidation state of the copper species is between valence states +1 and +2 in these samples, as indicated by the previous XPS results. Due to the reducibility of hydrogen, the valence state of Cu-600-H_2_ is more inclined toward Cu^0^, while the valence state of Cu-600-NH_3_ is almost +0.5 on average. The Fourier-transformed (FT) *k*3-weighted EXAFS oscillation in Fig. 2d and S15† showed that the three catalysts all demonstrate characteristic peaks similar to those of the Cu foil, with a prominent peak at a distance of approximately 2.1, corresponding to the Cu–Cu coordination in *R* space. Furthermore, scattering was detected at a distance of 1.5 Å, which corresponds to the first coordination shell of Cu–C in Cu-600-N_2_. This observation was further confirmed by the appearance of the Cu–C peak at 283.2 eV in the C 1s XPS spectrum, providing additional evidence for the coordination environment of the Cu–C bond in Cu-600-N_2_.

### CO_2_RR activity investigation

**Fig. 3:**
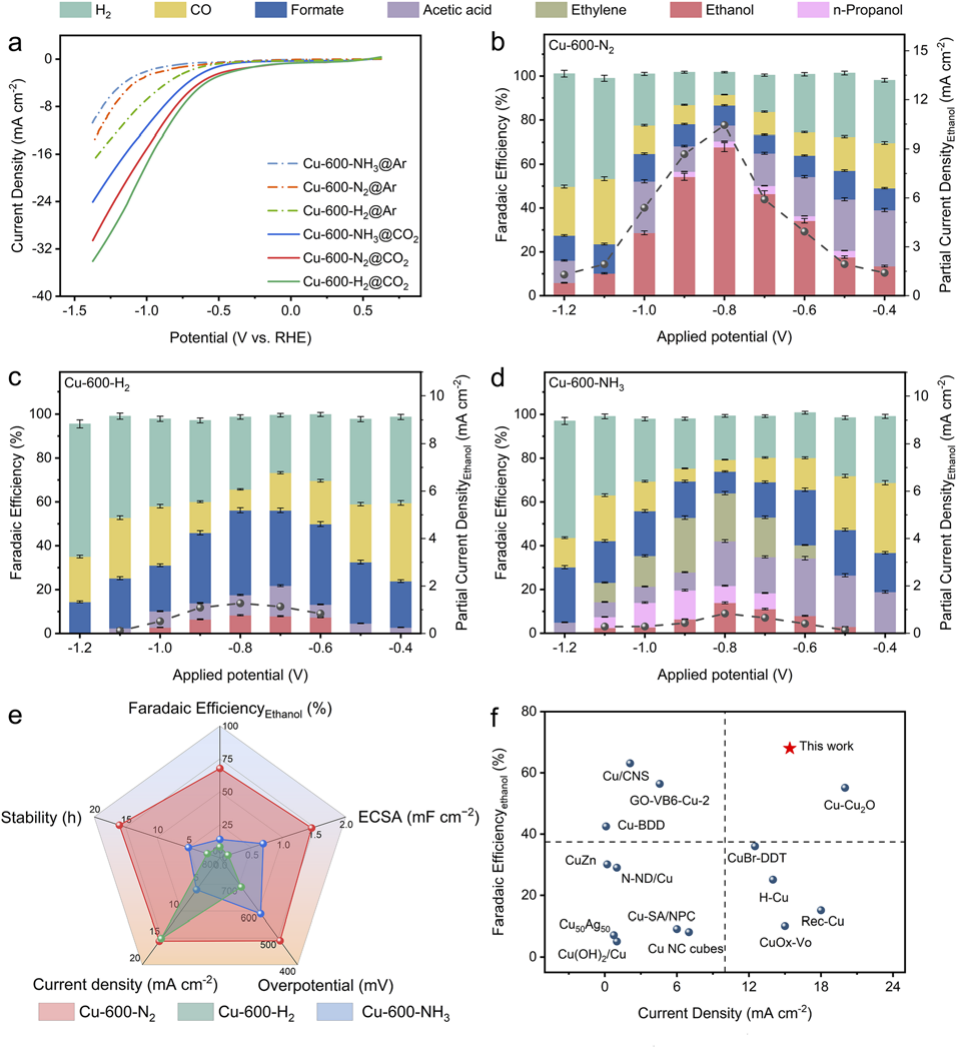
(a) LSV curves in CO_2_- and Ar-saturated 0.1 M KHCO_3_ aqueous solutions for Cu-600-N_2_, Cu-600-H_2_, and Cu-600-NH_3_. FE and partial current density for (b) Cu-600-N_2_, (c) Cu-600-H_2,_ and (d) Cu-600-NH_3_ in CO_2_-saturated 0.1 M KHCO_3_ aqueous solutions at different potentials (*vs.* RHE), and error bars are given by the shaded areas. The error bar indicates the standard deviation of three independent measurements. (e) Comparison of the overall performance of the three catalysts, Cu-600-N_2_, Cu-600-H_2,_ and Cu-600-NH_3_. (f) Comparison of stability with other recently reported advanced CO_2_RR Cu-based electrocatalysts in an H-type cell.

The CO_2_RR catalytic performances of Cu-600-N_2_, Cu-600-H_2_ and Cu-600-NH_3_ were evaluated in an H-type cell with a CO_2_-saturated 0.1 M KHCO_3_ electrolyte. Fig. 3a shows the linear sweep voltammetry (LSV) curves for different catalysts in CO_2_- and Ar-saturated 0.1 M KHCO_3_ electrolytes. The notably higher current density observed in a CO_2_-saturated electrolyte in comparison to an Ar-saturated electrolyte indicates the reduction of CO_2_. In contrast, the current in the Ar-saturated electrolyte predominantly originates from the hydrogen evolution reaction (HER). Notably, Cu-600-N_2_ exhibits a significantly higher current density, indicating that Cu-600-N_2_ is more conducive to the CO_2_RR rather than the HER.

At a low current density, Cu-600-N_2_ with a graphitic C shell produced a substantial amount of CO and acetic acid (Fig. 3b), while a small amount of ethanol was generated, indicating that C–C coupling of Cu-600-N_2_ was initiated at a lower overpotential. As the applied potential became more negative, the selectivity of CO and acetic acid decreased while the FE of ethanol increased, exhibiting a volcano-like trend. Notably, the conversion of CO_2_ to ethanol exhibited the highest FE of 67.8% at −0.8 V, much higher than that of Cu-600-H_2_, Cu-600-NH_3,_ and other previously reported Cu-based catalysts for converting CO_2_ to ethanol.^32–34^ According to previous reports, high selectivity for ethanol suggests a greater amount of surface-bound CO* or CHO*, which is a crucial step in forming C_2_ products.^35^ The number of other hydrocarbons produced was minor compared to that of ethanol; small amounts of n-propanol, acetic acid, and formate were generated with FE values of 2.8%, 7.8%, and 9.3%, respectively. The ^1^H NMR spectra of the products after the CO_2_RR are shown in Fig. S17.† However, the HER gradually became dominant at more negative potentials. Isotope-labelled ^13^CO_2_ was used as feed gas to trace the carbon source in ethanol production. The ^1^H-NMR analysis also confirmed that ethanol was produced from the CO_2_RR and not due to contaminants^52^ (Fig. S18†).

The FEs for Cu-600-H_2_ and Cu-600-NH_3_ were also determined for comparison (Fig. 3c and d). At 0.8 V *vs.* RHE, formate was the predominant product (38.6%) for Cu-600-H_2_, with an ethanol FE of 10.3%. Because severe Cu reconstruction hindered the realization of the synergy effect, it was difficult to enhance the ethanol product selectivity due to favorable HER. For the Cu-600-NH_3_ electrode, the FE of ethanol was only 15.2% at −0.8 V *vs.* RHE. When the proportion of amorphous carbon was high, the selectivity of ethanol was poor, and the hydrocarbon products were a complex mixture (C_2_H_4_: 24.9%; *n*-propanol: 8.1%; acetic acid: 20.4%; and formate: 9.9%). At the same potential, the partial current density of Cu-600-N_2_ was substantially greater than that of Cu-600-H_2_ and Cu-600-NH_3_. It should be noted that the onset potential for C_2_H_5_OH formation on Cu-600-N_2_ was approximately −400 mV (*vs.* RHE), corresponding to an overpotential of ∼490 mV, which was lower than the overpotentials observed for Cu-600-H_2_ (∼690 mV) and Cu-600-NH_3_ (∼590 mV) for the reduction of CO_2_ to C_2_H_5_OH (Fig. 3e). These results directly demonstrate the significance of developing an appropriate graphitization degree system to prevent reconstruction of Cu and promote high selectivity for ethanol production.

In addition, temperature is also a crucial factor influencing the graphitization degree of carbon materials. Therefore, a series of Cu-*x*-N_2_ catalysts were prepared by altering the Cu-MOF annealing temperature (*x* = 400, 500, 600, 700, 800 and 900 °C). At lower temperatures of 400 or 500 °C (Fig. S19a and b†), Cu NPs are fully encapsulated in a thicker carbon shell, indicating that the bulk MOF has not been effectively delaminated. Increasing the calcination temperature is beneficial for decomposing the metal–organic framework. At 600 °C (Fig. S19c†), the Cu-MOF structure is almost entirely delaminated into layered structures, with partial removal of Cu NPs from the graphene shell, resulting in the formation of a porous structure and graphene nanocages (inset of Fig. S19c†). During the calcination process, Ostwald ripening occurs, and metal nanoparticles tend to reduce the total surface energy by aggregating and forming larger particles, thereby reducing the interfacial surface energy between the particles. Therefore, as the temperature further increases from 700 °C to 900 °C (Fig. S19d–f†), Cu NPs melt and migrate, aggregating into larger particles. Additionally, with the increase of temperature, the metal particles break through the constraints of the carbon shell, resulting in even larger aggregation sizes. Indeed, larger metal particles can lead to a reduced electrochemical active surface area and an increase in electron transfer resistance, ultimately resulting in a decrease in catalytic reaction activity. As shown in Fig. S20,† the FE of ethanol follows a volcano pattern as the applied temperature was increased, reaching a maximum of 67.8% at 600 °C, and the current reached 15.4 mA cm^−2^. The catalyst performance of the other two catalysts, Cu-*x*-H_2_ and Cu-*x*-NH_3,_ is also shown in Fig. S21 and S22.†

To explore the origins of enhanced CO_2_RR by different carbon shell structures, the influence of various catalysts on the active sites and charge transfer kinetics is examined. The electrochemical active surface area (ECSA) of the catalysts was estimated by measuring the electrochemical double-layer capacitance (Cdl) obtained from the CV curves at different scan rates.^36^ As shown in Fig. S23,† Cu-600-N_2_ can provide the largest ECSA, which facilitates an improved contact interface with the electrolyte and exposes more active sites to enhance the adsorption of various intermediates. The electrochemical impedance spectroscopy (EIS) depicted in Fig. S24† revealed that Cu-600-N_2_ exhibits a smaller semicircular radius and charge transfer resistance in the Nyquist plot, indicating a faster charge transfer process occurring on Cu-600-N_2_ compared to Cu-600-H_2_ and Cu-600-NH_3_. It expedites the transfer of electrons to CO_2_ to stabilize the reduced CO_2_˙ intermediate, which is essential for the electroreduction of CO_2_.^37,38^ Due to poor graphitization, the Cu-600-NH_3_ electrode features the most significant charge transfer resistance and the worst conductivity.^27^ This phenomenon demonstrates that a suitable degree of graphitization and a high Cu–C combination is advantageous for the CO_2_RR and that these two parameters may act synergistically to enhance the activity and selectivity of the CO_2_RR.

**Fig. 4:**
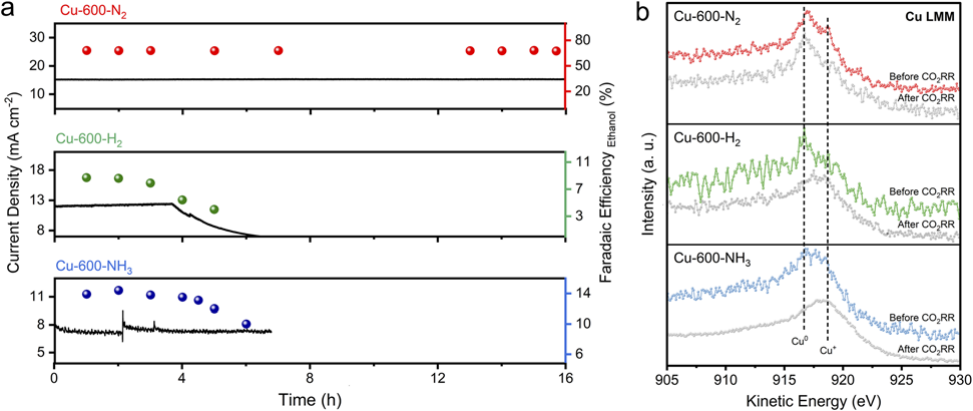
(a) CO_2_ electroreduction stability of Cu-600-N_2_, Cu-600-H_2,_ and Cu-600-NH_3_ at a potential of −0.8 V (*vs.* RHE) in 0.1 M KHCO_3_. (b) XPS Cu LMM spectra of Cu-600-N_2_, Cu-600-H_2,_ and Cu-600-NH_3_ before and after the CO_2_RR. The CO_2_RR was conducted at a potential of −0.8 V (*vs.* RHE) for 3 hours.

The stability of catalysts for the CO_2_RR is of utmost importance for their practical application. As shown in Fig. 4a, Cu-600-N_2_ remained stable and had excellent catalytic activity over 16 hours at an applied potential of −0.8 V, which is more stable than the other catalysts. SEM revealed that the C shell-coated Cu nanoparticles maintained their embedded morphology and chemical state even after long reaction times (Fig. 4b and S25†). Cu wrapped by a protective shell exhibited significantly improved stability due to the prevention of Cu particle aggregation. For unprotected Cu nanoparticles or disordered carbon, however, the surface changed significantly after a few hours at a high reaction rate (Fig. S26–S28†). The Cu nanoparticles on the surface underwent severe surface reconstruction and agglomerated into many micron-sized and rough aggregates, and the ethanol selectivity also decreased rapidly. Furthermore, as depicted in Fig. S29,† the anti-corrosion properties of Cu-600-N_2_, Cu-600-H_2_, and Cu-600-NH_3_ were evaluated by Tafel analysis. Cu-600-N_2_ exhibited the best corrosion resistance among the three catalysts due to its more positive corrosion potential than Cu-600-H_2_ and Cu-600-NH_3_.

The above performance comparison demonstrates that Cu-600-N_2_ exhibited superior performance to Cu-600-H_2_ and Cu-600-NH_3_ in multiple aspects (as depicted in Fig. 3e). The outstanding stability of Cu-600-N_2_ could be attributed to the protective effect of the carbon layer encapsulating the Cu nanoparticles. The internal Cu nanoparticles could also collaborate with the surface carbon shell to form stable Cu–C bonds, promoting the catalytic reaction and producing a synergy effect.^39–41^ This synergistic effect enabled Cu-600-N_2_ to maintain high FE and current density during continuous electrocatalysis, making it comparable to recently reported Cu-based electrocatalysts for CO_2_-to-ethanol conversion in an H-cell setup (as shown in Fig. 3f and Table S4†).

### Mechanistic study on enhanced C–C coupling

**Fig. 5:**
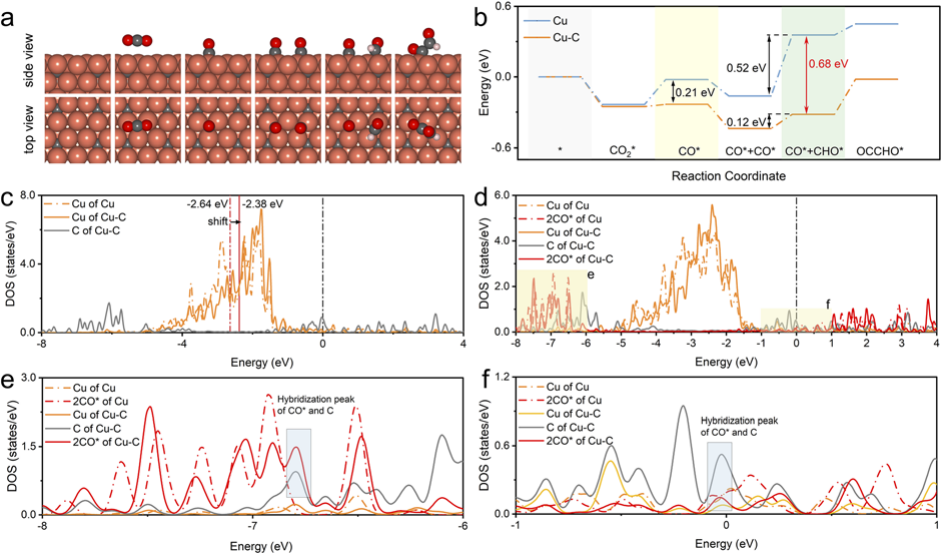
(a) The proposed dimerization mechanism for ethanol formation involves optimized structures of adsorbed species on the Cu–C surface, shown in the top and side views. Cu: orange, C: gray, O: red, H: white. (b) Free energy diagram for CO dimerization on the pristine Cu–C and bare Cu surface. (c) The calculated d-PDOS of surface Cu and C atoms on Cu–C and bare Cu planes. The two blue lines indicate the d-band center positions with the calculated values. (d) The d-PDOS of surface Cu, C, and CO* on Cu–C and bare Cu planes after adsorption of the two intermediate CO*, and (e) and (f) are the enlarged local views.

The experiment confirmed that the carbon coating of the Cu nanoparticles played a significant role in ethanol generation. To gain more fundamental insights, DFT calculations were also performed to verify the effect of the Cu–C bond on the performance of the CO_2_RR to ethanol. The Cu–C bond structure model was constructed by doping C on the subsurface of Cu, while bare Cu was used for comparison. The doping sites of C and the adsorbed sites are based on the most energetically stable structures. Fig. 5a illustrates the schematic diagrams for CO_2_RR pathways that achieve CO* and CO* dimerization on Cu–C. For bare Cu surfaces, the schematic diagrams of the reaction pathway are illustrated in Fig. S30.† Initially, one CO_2_ molecule forms a *CO species on the copper surface through protonation, and then *CHO is formed by one proton transfer process. Subsequently, *CHO undergoes coupling with another CO* by *CHO + CO* → *CHO–CO,^42,43^ which is considered the rate-determining step in the ethanol formation path. The free energy profiles (Fig. 5b) suggest that Cu–C exhibits a low reaction energy barrier (0.02 eV) in the process of CO* formation, significantly lower than that on bare Cu (0.21 eV). Therefore, the Cu–C surface is favorable for the formation of CO*, which can improve the coverage of CO*. It has been reported that the energy barrier for CO dimerization can be lowered by increasing the coverage of CO*, which can improve its dimerization.^44,45^ When CO is further hydrogenated to generate CHO, the energy barrier is as low as 0.12 eV on the Cu–C surface, which reduces the overall reaction energy barrier for subsequent C–C coupling. In addition, the uphill energy for CHO formation on bare Cu is 0.52 eV, which is much higher than that on Cu–C (0.4 eV). The large free energy implies high-energy barriers for the CO_2_-to-C_2_ process. Therefore, the Cu–C bond can promote the CO_2_RR to form CO* and OCCHO* and reduce the energy barrier for CO* dimerization, which leads to higher selectivity for ethanol.

In addition, the charge density difference (CDD) of Cu–C was calculated to study the space charge distribution and influence on the valence state of the Cu and electric properties of the catalyst. Fig. S31–S33† shows that based on DFT calculations, Cu–C exhibits charge transfer from Cu to carbon due to the strong electronic attraction properties of the graphic structure; this was consistent with the XPS fitting results in the experiment. The plots of the d-projected density of states (d-PDOS) of Cu and Cu–C in the unabsorbed state show that the carbon effect upshifts the d-band center of the surface Cu from −2.64 to −2.38 eV, indicating enhanced activity of surface Cu atoms (Fig. 5c). This electronic interaction results in the d orbital electron of Cu atoms being relatively empty, facilitating electron coupling with the p orbital electron of CO_2_ adsorbates. Moreover, doped C displays peaks near the Fermi level, which enables electrons with energies close to the Fermi level to participate in bonding during adsorption, thus enhancing the interaction with the adsorbate. As displayed in Fig. 5d–f, comparing adsorption configurations of two CO* reveals a noticeable rightward shift of the d-band of C-doped Cu. The solid red line in the energy range of −8 eV to −6 eV and near the Fermi level shows hybridization between Cu, C, and the adsorbed CO*, indicating that doped C can also hybridize with the electrons of adsorbed CO*, further enhancing the interaction with the catalyst.^46^

## Conclusion

In summary, we have designed a highly efficient and stable electrocatalyst, Cu-600-N_2_ by forming a carbon shell on Cu nanoparticles for CO_2_ electroreduction to ethanol. First, the carbon shell could form Cu–C bonds with Cu nanoparticles, thereby tuning the electron environment around Cu atoms and promoting the formation and coupling process of CO*. At the same time, the carbon shell improved the structural and chemical stability of the catalyst. DFT calculations showed that Cu–C bonds regulated the key intermediate *HOCCH's hydrogenation pathway and favored the ethanol formation reaction pathway. Therefore, the catalyst exhibited a high ethanol FE of 67.8% and excellent stability. Our results and conclusions pave the way for the rational design of efficient and stable catalysts for the CO_2_RR.

## Author contributions

Y. T. and W. X. proposed the project, designed the experiments and wrote the manuscript; Y. T. performed the whole experiments; S. T. H., X. D., S. Q. J., M. W., D. W. Z. C. J. C. and J. H. Y. assisted in analyzing the experimental data; S. Q. J., W. X., X. Q. X., M. Y. H. and H. H. W. assisted in analyzing the experimental data of XAS; B. X. H. supervised the whole project.

## Conflicts of interest

The authors declare no competing interests.

## Supplementary Material

SC-014-D3SC04061E-s001
