# Supplementary material for: Optimizing copper nanoparticles with a carbon shell for enhanced electrochemical CO2 reduction to ethanol
Source: Chem Sci. 2023 Nov 24;14(48):14308–15. doi: 10.1039/d3sc04061e (PMC10718077; doi:10.1039/d3sc04061e)
Supplement: SC-014-D3SC04061E-s001 [file SC-014-D3SC04061E-s001.pdf]

Electronic Supplementary Material (ESI) for Chemical Science.
This journal is © The Royal Society of Chemistry 2023

Optimizing Copper Nanoparticles with Carbon Shell for Enhanced Electrochemical CO_2 Reduction to Ethanol

Ting Yao[a], Wei Xia[a]*, Shitao Han[a], Shuaiqiang Jia[a], Xue Dong[a], Min Wang[a], Jiapeng Jiao[a], Dawei Zhou[a], Jiahao Yang[a], Xueqing Xing[d], Chunjun Chen[a], Mingyuan He[a,c], Haihong Wu[a,b]*, Buxing Han[a,b,c]*

Abstract: The electrochemical reduction of carbon dioxide (CO_2RR) holds great promise for sustainable energy utilization and combating global warming. However, progress has been impeded by challenges in developing stable electrocatalysts that can steer the reaction toward specific products. This study proposes a carbon shell (C shell) coating protection strategy by an efficient and straightforward approach to prevent electrocatalyst reconstruction during CO_2RR. Utilizing a Copper-based Metal-Organic Framework (Cu-MOF) as the precursor for the C shell, we synthesized carbon shell-coated electrocatalysts, denoted as Cu-*x*-*y*, through calcination in an N_2 atmosphere (where *x* and *y* represent the differ calcining temperature and atmosphere: N_2, H_2, and NH_3). It was found that the Faradaic Efficiency (FE) of ethanol over the catalysts with a C shell could reach ~67.8%. In addition, the catalyst could be stably used for more than 16 h, surpassing the performance of Cu-600-H_2 and Cu-600-NH_3. Control experiments and theoretical calculations revealed that the carbon shell and Cu-C bonds played a pivotal role in stabilizing the catalyst, tuning the electron environment around Cu atoms, and promoting the formation and coupling process of CO*, ultimately favoring the reaction pathway leading to ethanol formation. This carbon shell coating strategy is valuable for developing highly efficient and selective electrocatalysts for CO_2RR.

Experimental Procedures

Chemicals and reagents

All chemicals were of analytical grade and directly used as purchased without any further purification. Copper nitrate trihydrate ($Cu(NO_3)_2 \cdot 3H_2O$), Terephthalic acid (H_2BDC), 1, 4-diazabicyclooctane (DABCO) were obtained from Aladdin (Shanghai, China). N, N-dimethylformamide (DMF) and Ethanol (EtOH) were purchased from Guoyao Chemical Co. Ltd.(Shanghai, China). All aqueous solutions were prepared using ultrapure water.

Characterization

The obtained samples were analyzed by multiple test methods. X-ray diffraction (XRD) was performed on a Bruker D8 tools X-ray diffractometer with Cu Kα radiation source (λ =0.154 nm) operating at 40 kV and 30 mA. The content of metals in the catalysts was determined by inductively coupled plasma optical emission spectroscopy (ICP-OES, Optima 8300, Perkin-Elmer). Nitrogen adsorption-desorption measurement was carried out at 77 K on Autosorb-1 (Quantachrome USA, Inc.), and the specific surface areas, microporous surface area, and pore size distribution were calculated based on the Brunauer–Emmett–Teller method, t-plot method (P/P0=0.27-0.40), and the nonlocal density functional theory method, respectively. Ex-situ Raman spectra were recorded on a RENIDHAW via Raman microscope with a 532 nm argon ion laser. X-ray photoelectron

spectroscopy (XPS) tests were performed on a Thermo Fisher Microlab 350 photoelectron spectrometer with Al Kα X-ray beam, and all of the binding energies were calibrated according to the C 1s peak centered at 284.6 eV. Scanning electron microscopy (SEM) images were obtained on a Zeiss S450 microscope at 5 kV. Transmission electron microscopy (TEM) images were obtained by using a JEOL F200 with an image corrector, and the mapping images were acquired from energy-dispersive X-ray spectra in scanning transmission electron microscopy (STEM) mode. The X-ray absorption fine structure spectra data were collected at the 4B9A beamline in Beijing Synchrotron Radiation Facility, China (BSRF, operated at 2.5 GeV with a maximum current of 250 mA). The data were obtained in fluorescence excitation mode using a Lytle detector. The normalization of data was accomplished by subtracting the edge and post-edge backgrounds using the Athena program, in which the edge jump was assigned to 1.0.

Synthetic procedures for Cu-MOF

The Cu-MOF was prepared using a solvothermal method. First, 0.2 mmol (28.9 mg) of $Cu(NO_3)2\cdot 2.5H_2O$, 0.2 mmol (32.4 mg) of H_2BDC, and 0.1 mmol (11.2 mg) of DABCO were sequentially added to 40 mL of N,N-dimethylformamide (DMF) and sonicated for 15 minutes to dissolve them. Subsequently, the resulting blue solution was transferred into a Teflon liner and stirred for 10 minutes. The mixture was then placed into a stainless-steel autoclave of 100 mL and heated at 120°C for 12 hours. After cooling to room temperature, the solid green product, Cu-MOF, was obtained through centrifugation and freeze-drying.

Synthetic procedures for Cu-600-N_2 and other catalysts

Cu-MOF was placed in a tube furnace and continuously purged with nitrogen gas. The temperature was gradually increased at a rate of 2°C per minute until reaching 600°C, and held at this temperature for 2 hours for the annealing process. Upon cooling to room temperature, the final desired catalyst, denoted as Cu-600-N2, was obtained. By changing the final annealing temperatures to 400, 500, 700, 800, and 900, a series of Cu-x-N_2 catalysts (where x represents the annealing temperature) were prepared.

Similarly, for the fabrication of Cu-x-H_2 and Cu-x-NH_3, analogous procedures were employed, but with the introduction of hydrogen gas (H_2) and ammonia gas (NH_3), respectively, in place of nitrogen.

Preparation of the Working Electrode

5 mg of catalyst powder was mixed with 20 μL of 5% Nafion and 1 mL of isopropanol solution, and sonicate for one hour to achieve uniform dispersion, obtaining the catalyst ink. Subsequently, 70 μL of the catalyst ink was dropped onto a 1 cm^2 carbon paper and dry to obtain the catalyst working electrode.

Electrochemical study

Electrochemical experiments were carried out on CHI instruments model 760E electrochemical workstation using an H-cell reactor with two compartments and a proton-exchange membrane. The electrolyte was 0.1M $KHCO_3$ aqueous solution (pH = 6.5). The measurements were performed using iR compensation. Before CO_2 reduction, the electrolyte was injected into each compartment and purged with CO_2 gas for 30 minutes. Using a digital gas

flow controller, the flow rate of CO_2 gas through the gas chamber was set at 15 sccm. A coiled platinum wire served as the counter electrode, and an Ag/AgCl (3 M KCl saturated) electrode acted as the reference electrode. First, the working electrode was electrochemically reduced for five cycles using cyclic voltammetry (CV) between 0.5 and 2.0 V versus RHE at a rate of 0.1 V s^{-1}. CO_2RR was measured for 30 minutes using an i-t curve at each fixed potential. Gas chromatography (Agilent 7890 GC) was used to detect gas products after the gas was collected with a gas bag. 1H nuclear magnetic resonance (NMR Bruker AVANCE III HD) was used to quantify liquid products. So, after electrolysis, 1000 μL of electrolyte was mixed with 100 μL of 12.5 mM phenol and 10 mM DMSO as reference. After shaking, 400 μl of the mixture was added to 200 μL of deuterated water (D_2O). The following equation was employed to convert the potential to RHE: $E_{RHE} = 0.0591\ pH + 0.197\ V + E_{Ag/AgCl}$; The following equation was used to calculate the FE for the products: $FE(\%) = z \times F \times n/Q \times 100 = z \times F \times n/(I \times t) \times 100$, where z is the number of electrons transferred, F is the Faraday constant, Q is the total transferred charge, I is the current, t is the running time and n is the total amount of the product (in mole). The ECSA double-layer capacitance was measured by CV in a 0.1 m $KHCO_3$ electrolyte, which was estimated by the slope of the linear fits and was proportional to the ECSA. Electrochemical impedance spectroscopy (EIS) data were recorded with the frequency range of 0.01~100000 Hz.

DFT calculations

All calculations were executed by using the projector augmented wave method according to the density functional theory (DFT), which could be realized by Vienna ab-initio Simulation Package (VASP) [1-3]. All geometric optimizations were carried out with the Perdew–Burke–Ernzerhof (PBE) exchange and correlation energy within generalized gradient approximation (GGA) [4,5]. The plane-wave energy cutoff was set to 400 eV. The Brillouin-zone sampling was conducted using Monkhorst-Pack (MP) grids of special points with a separation of 0.04 $Å^{-1}$. The self-consistence-field convergence criterion was set to 10^{-4} eV, and the atomic positions were optimized with the convergence criterion of 0.05 eV/Å. Gaussian smearing of 0.05 eV was applied to speed up self-consistent field convergence. The 5-layer 4×4 supercell Cu(111) slab was used to build the Cu and Cu-C model. A vacuum height of 15 Å along the vertical direction was selected to avoid the unwanted interaction between the slab and its period images. The final structure was illustrated with VESTA software [6].

1 G. Kresse, J. Hafner, *Phys. Rev. B* **1993**, *47*, 558–561.

2 G. Kresse, J. Furthmüller, *Phys. Rev. B* **1996**, *54*, 11169–11186.

3 G. Kresse, J. Furthmüller, *Computational Materials Science* **1996**, *6*, 15–50.

4 J. P. Perdew, K. Burke, M. Ernzerhof, *Phys. Rev. Lett.* **1996**, *77*, 3865–3868.

5 J. P. Perdew, Y. Wang, *Phys. Rev. B* **1992**, *45*, 13244–13249.

6 K. Momma, F. Izumi, *Journal of Applied Crystallography* **2011**

Results and Discussion

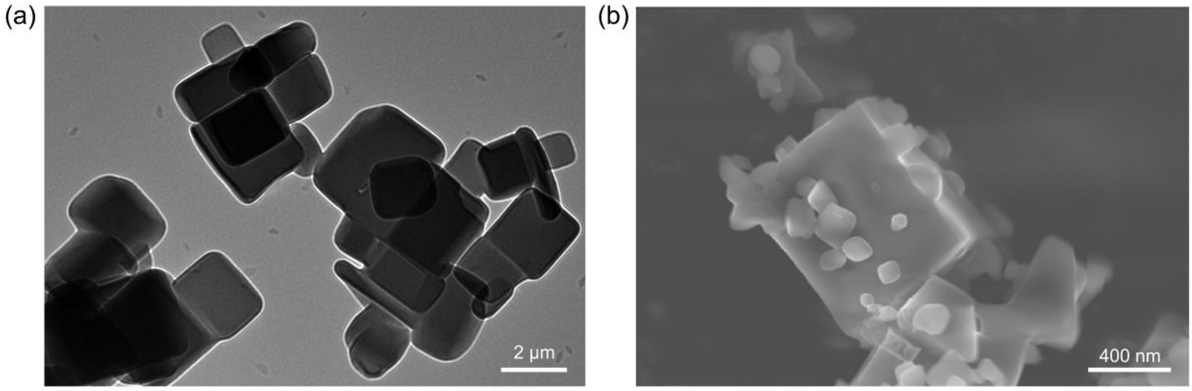

Figure S1. The (a) TEM and (b) SEM images of the Cu-MOF.

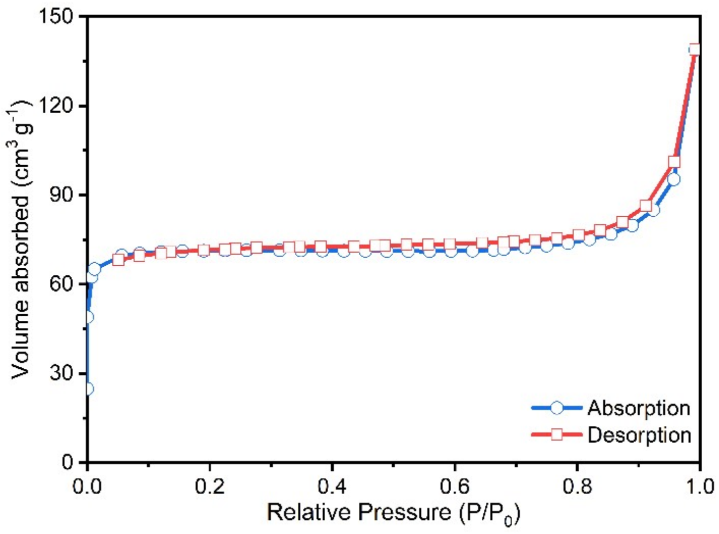

Figure S2. N_2 adsorption and desorption isotherms of the Cu-MOF at 77 K.

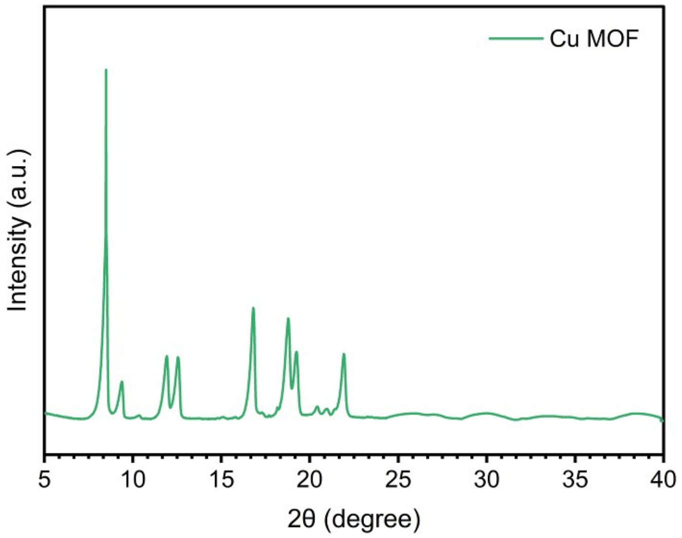

Figure S3. XRD patterns of the Cu-MOF precursor.

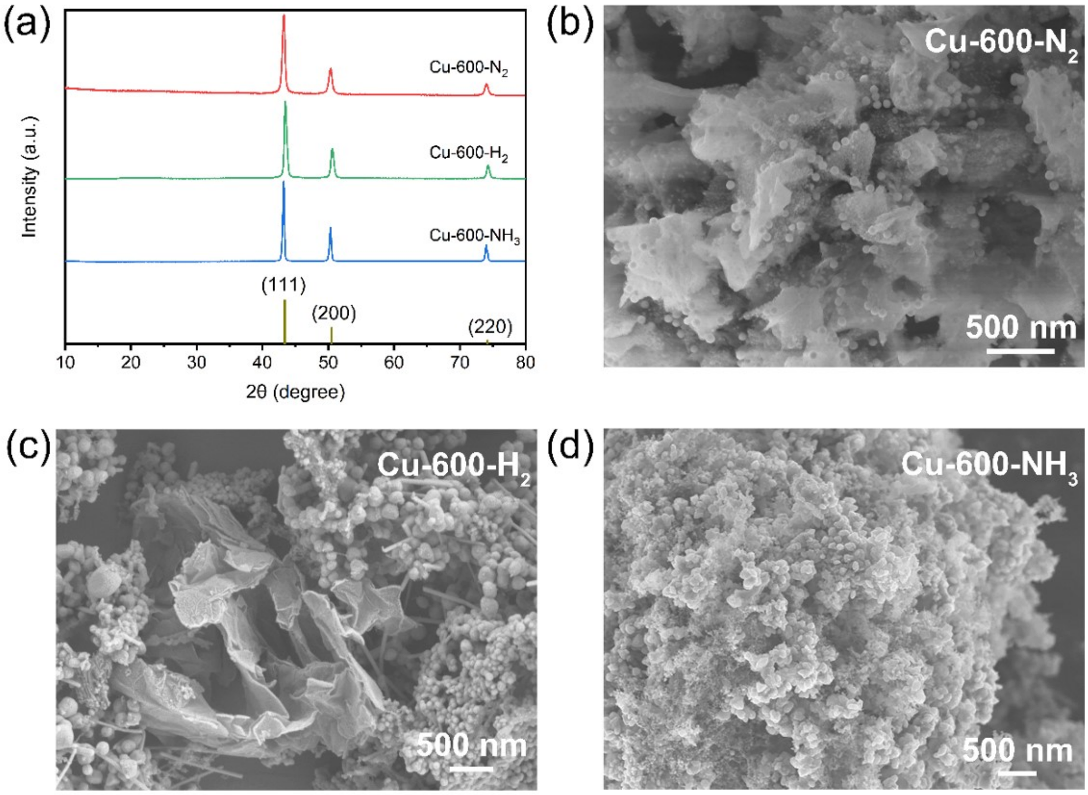

Figure S4. (a) XRD patterns of Cu-600-NH_3, Cu-600-N_2, Cu-600-H_2. Surface structure of the (b) Cu-600-N_2 (c) Cu-600-H_2, and (d) Cu-600-NH_3, respectively. The copper nanoparticles of Cu-600-N_2 are coated with a thin layer of carbon shell on the surface. The Cu nanoparticles in Cu-600-H_2 are separated from carbon. The Cu nanoparticles in Cu-600-NH_3 are surrounded by a fragmented carbon layer.

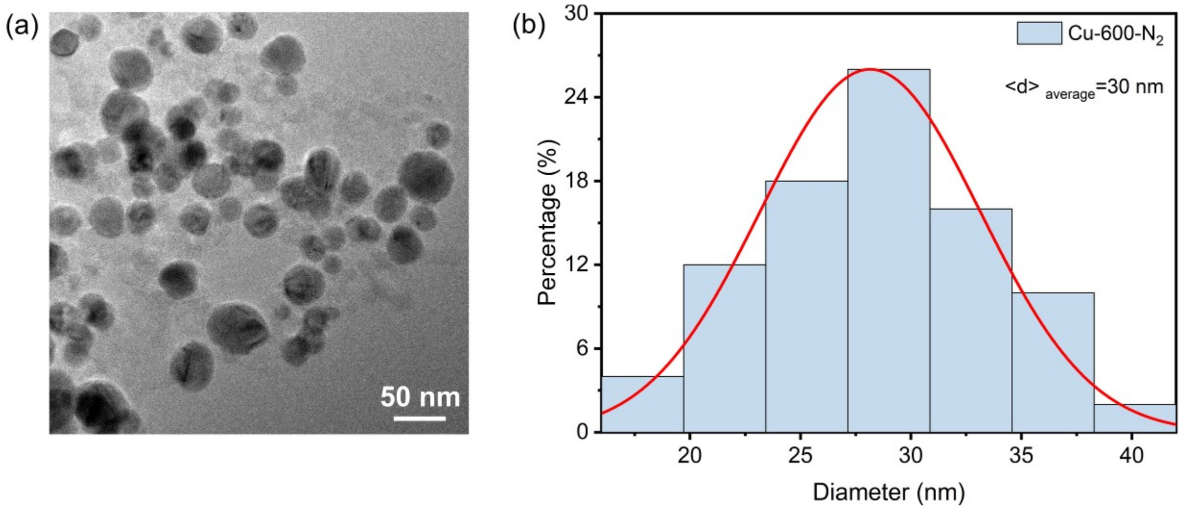

Figure S5. (a) A typical TEM characterization for catalyst Cu-600-N_2. (b) The particle-size distribution from TEM imaging reveals an average particle size of 30 nm for Cu-600-N_2.

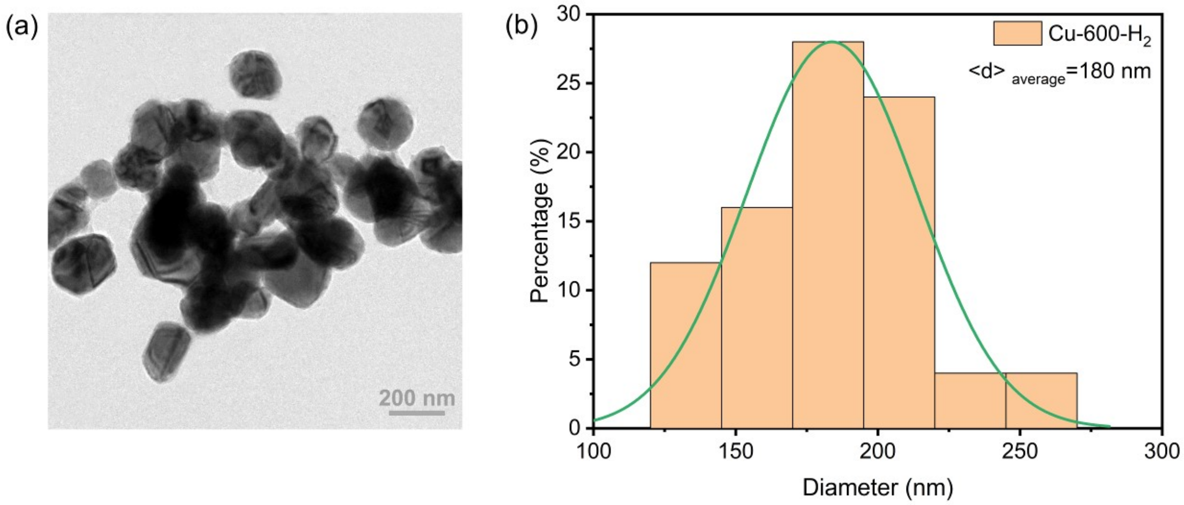

Figure S6. (a) A typical TEM characterization for catalyst Cu-600-H_2. (b) The particle-size distribution from TEM imaging reveals an average particle size of 180 nm for Cu-600-H_2.

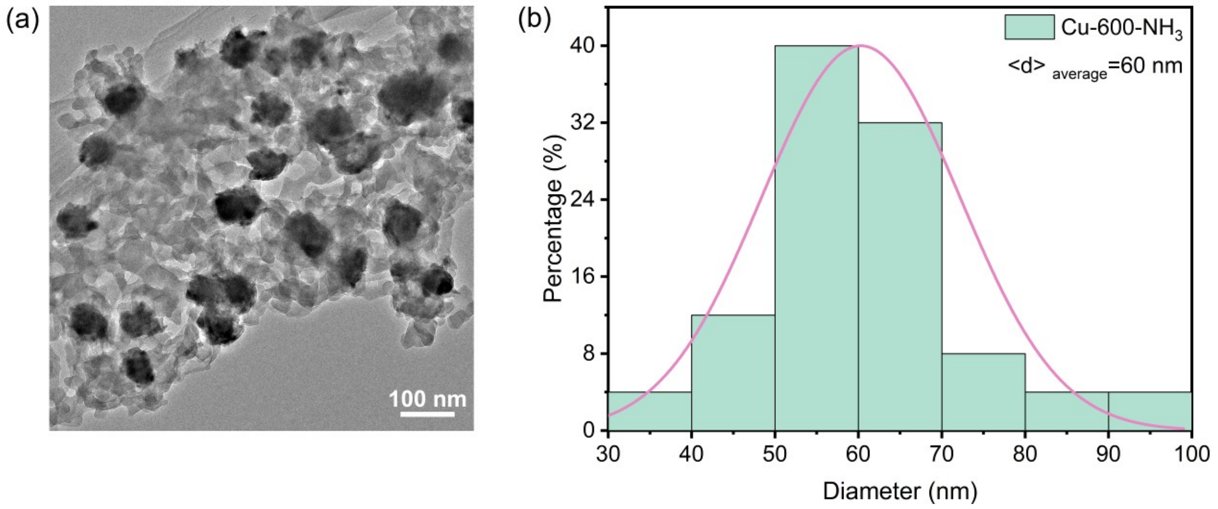

Figure S7. (a) A typical TEM characterization for catalyst Cu-600-NH_3 (b) The particle-size distribution from TEM imaging reveals an average particle size of 60 nm for Cu-600-NH_3.

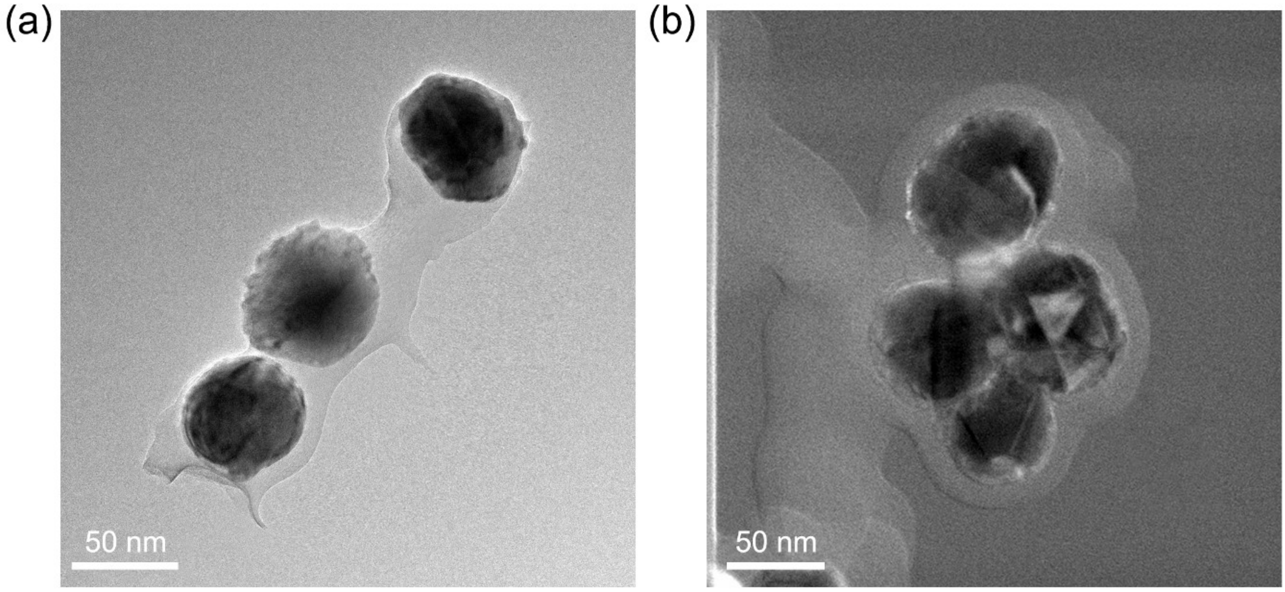

Figure S8. (a) TEM and (b) STEM images of copper nanoparticles with carbon shell coating in sample Cu-600-N_2 after half an hour of ultrasonic.

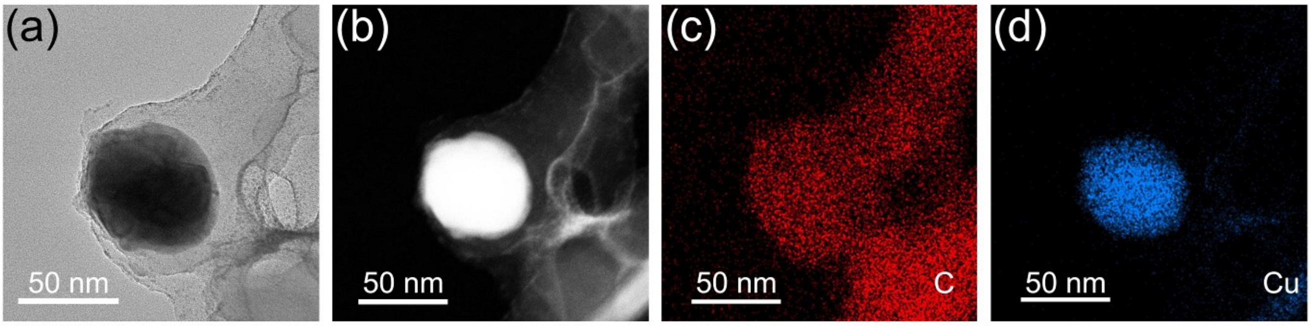

Figure S9. EDS mapping image of Cu-600-N_2 (a) STEM, (b) TEM, (c) C, and (d) Cu. There is a significant carbon aggregation near the Cu nanoparticles.

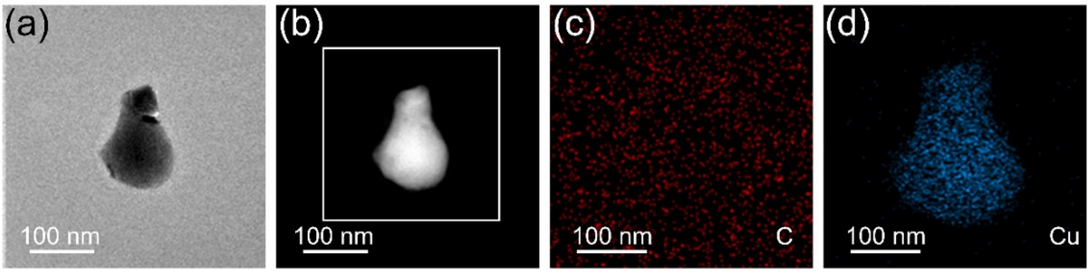

Figure S10. EDS mapping image of Cu-600-H_2 (a) STEM, (b) TEM, (c) C, and (d) Cu. STEM and element mapping indicate that there is no C aggregation around Cu.

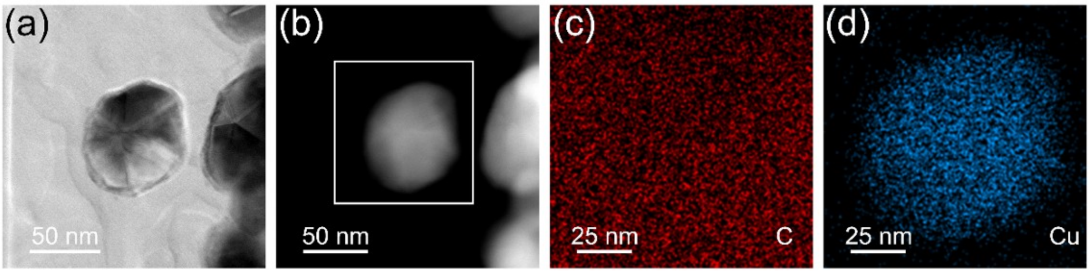

Figure S11. EDS mapping image of Cu-600-NH_3 (a) STEM, (b) TEM, (c) C, and (d) Cu. The carbon around the Cu nanoparticles is uniformly dispersed and does not form a tightly bound structure with Cu.

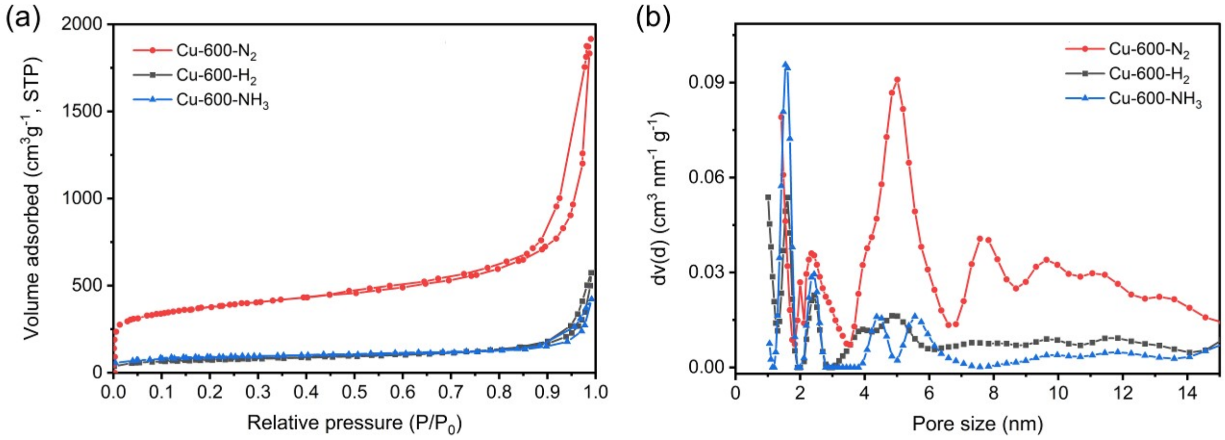

Figure S12. (a) N_2 adsorption-desorption isotherms and (b) pore size distribution of Cu-600-N_2, Cu-600-H_2, and Cu-600-NH_3 respectively.

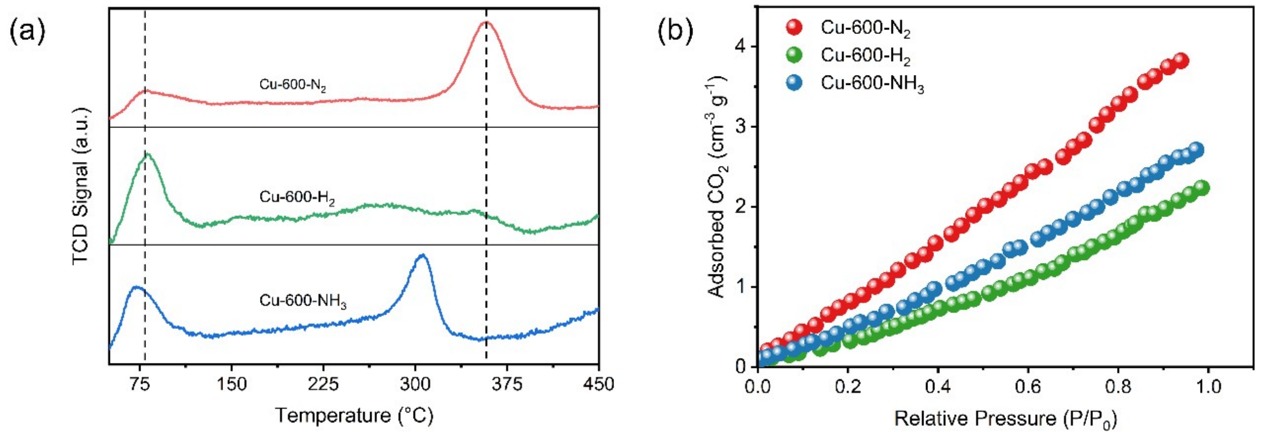

Figure S13. (a) CO_2-TPD profiles for Cu-600-N_2, Cu-600-H_2 and Cu-600-NH_3 from room temperature to 450°C. (b) CO_2 adsorption isotherms at 25 °C for Cu-600-N_2, Cu-600-H_2, and Cu-600-NH_3.

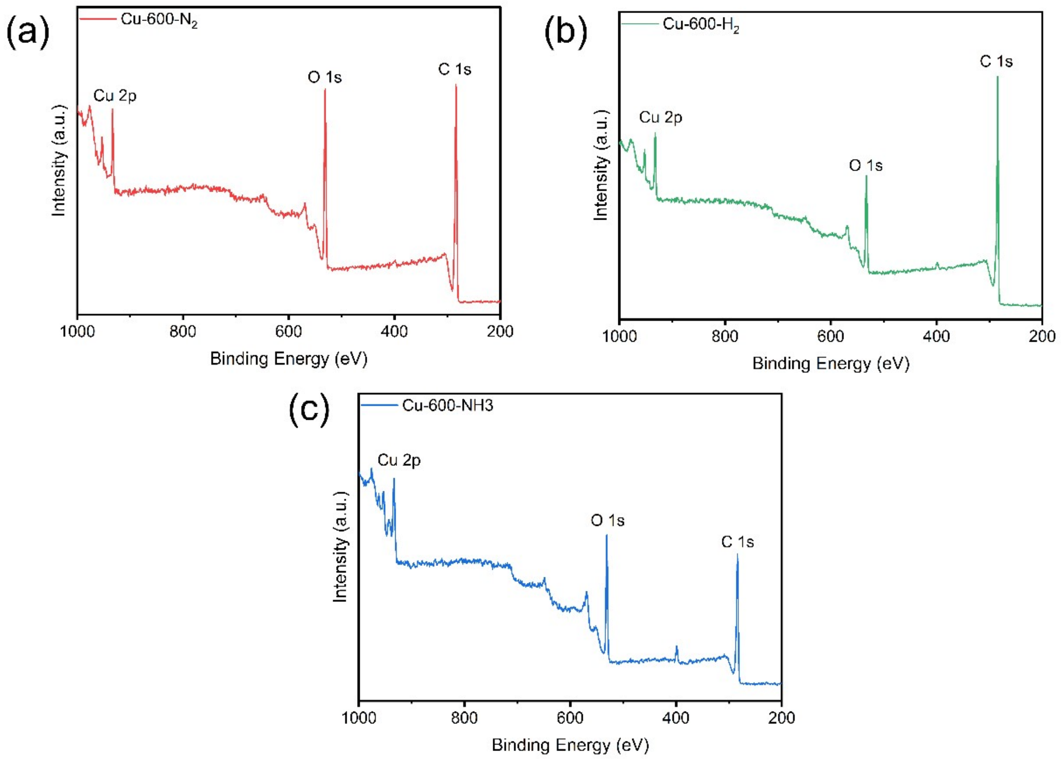

Figure S14. The XPS survey spectrum of Cu-600-N_2, Cu-600-H_2 and Cu-600-NH_3.

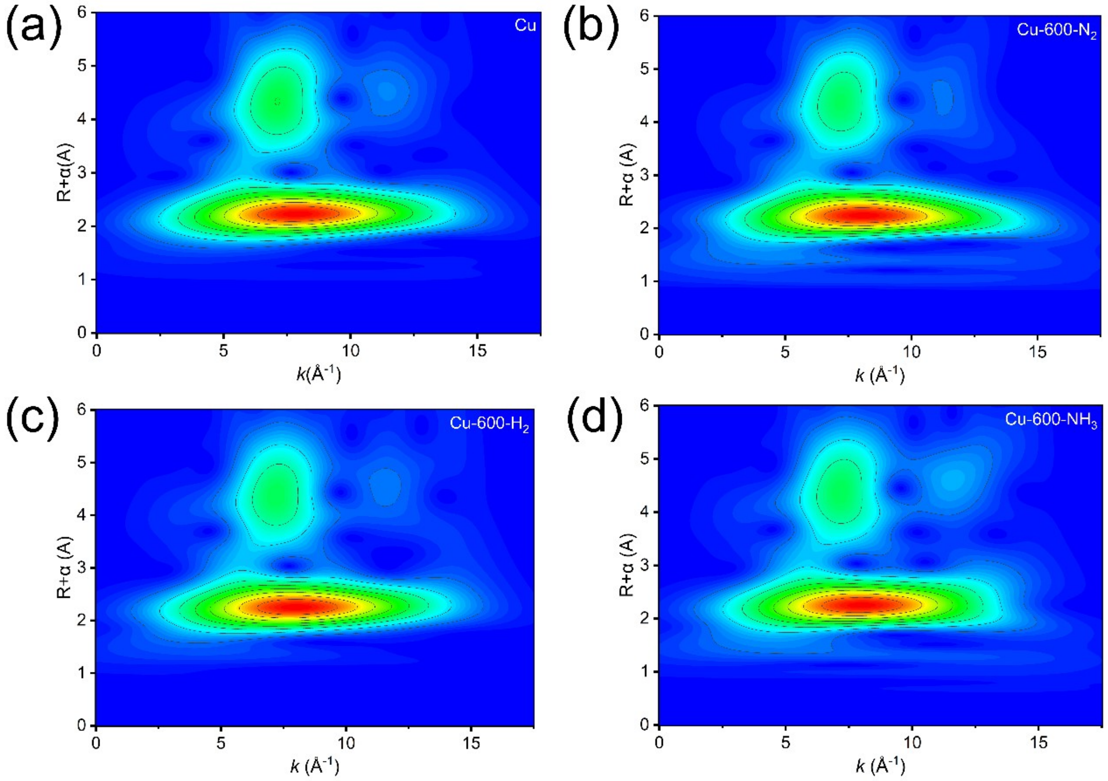

Figure S15. Morlet WT of the *k*3-weighted EXAFS data for Cu, Cu-600-N_2, Cu-600-H_2, Cu-600-NH_3.

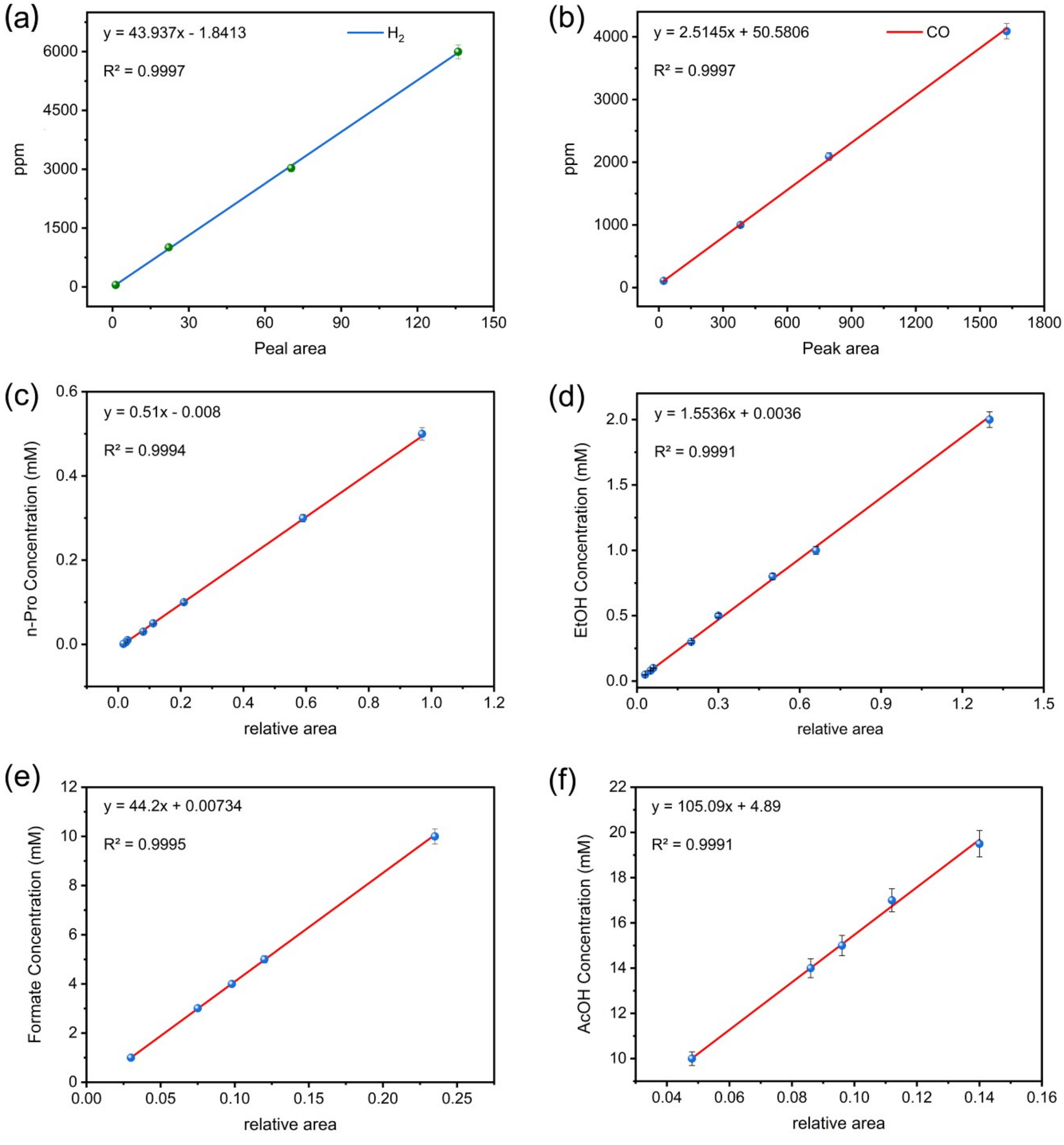

Figure S16. Standard curves for H_2 (a) and CO (b) products for GC analysis. The standard curves were constructed using known standard concentration gas samples. The standard curves for (c) n-Propanol, (d) Ethanol, (e) formate, and (f) acetic acid. The standard curves were made using standard chemicals over the concentration range of interest, with the internal standard phenol and DMSO. The linearity of the curves is as high as 0.999.

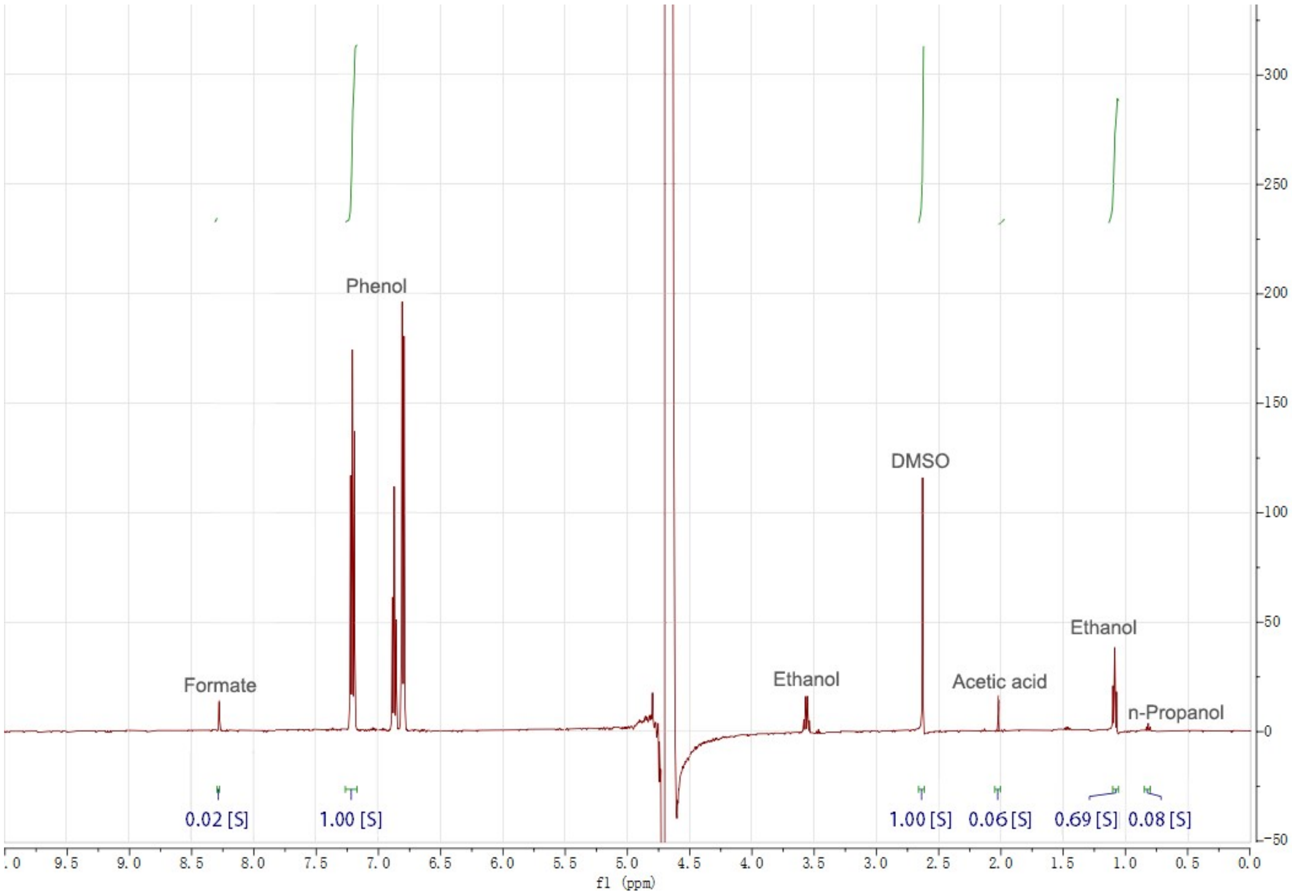

Figure S17. ^{1}H NMR spectra of the electrolyte taken after 1h electrocatalysis over Cu-600-N_2 at –0.8 V vs RHE. After the 1h electrolysis at −0.8 V vs RHE, the ratios of the areas of the produced acetate, n-propanol at 0.9 ppm and EtOH at 1.17 ppm to the DMSO peak area and formate to the phenol peak area were calculated, respectively. The obtained ratios were then compared to the standard curves to quantify the concentrations of the reaction products.

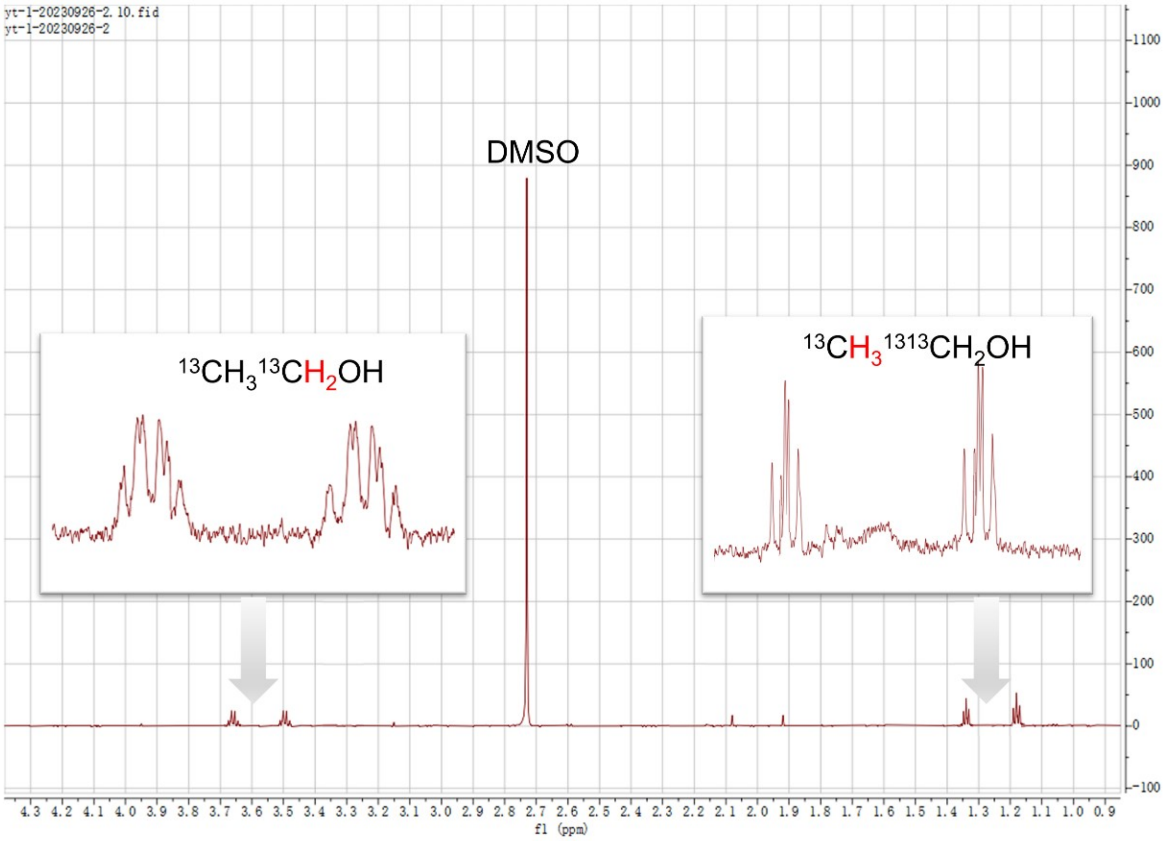

Figure S18. ^{1}H-NMR spectra of the electrolyte after 1 h $^{13}CO_2$ electroreduction at -0.8 V on the Cu-600-N_2 in 0.1 M $KHCO_3$, and inserts is the corresponding enlarged ^{1}H-NMR spectra.

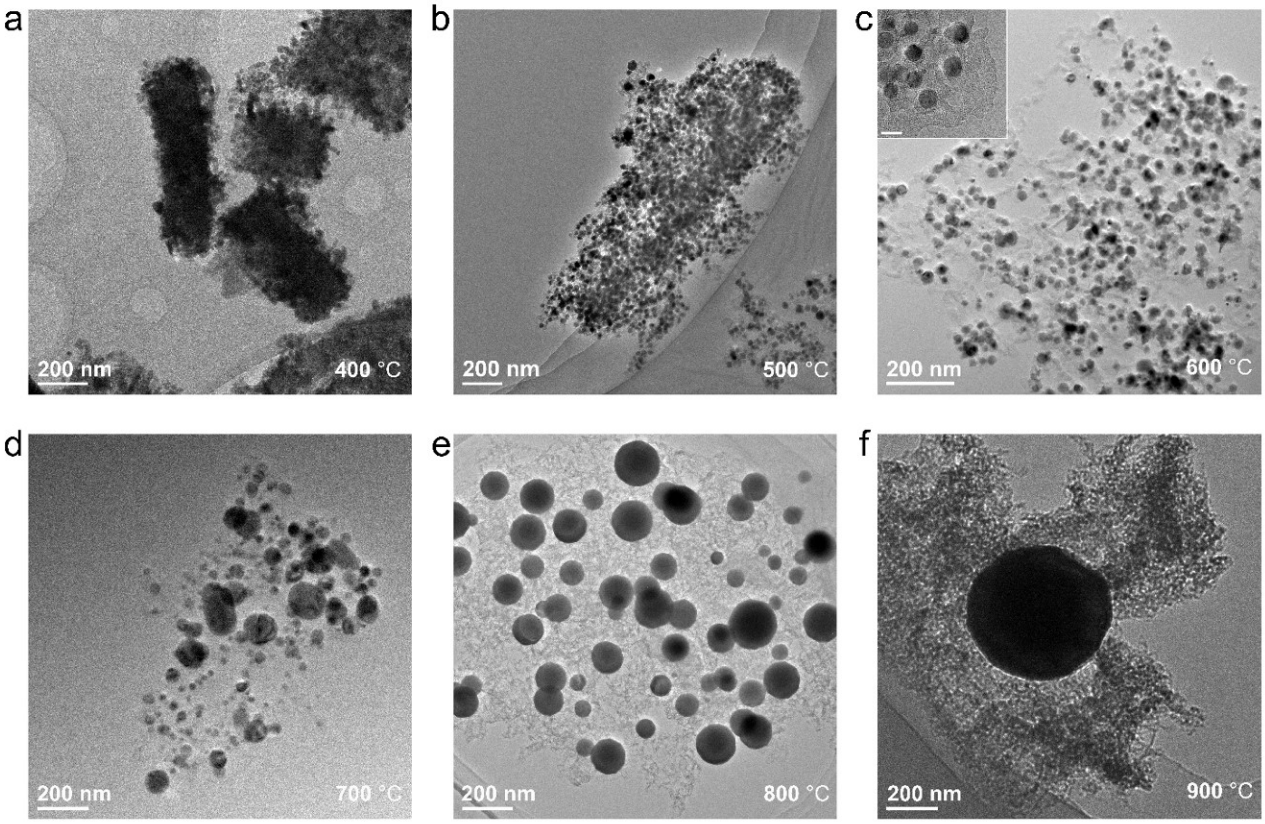

Figure S19. TEM images of the Cu-MOF precursor calcined at 400, 500, 600, 700, 800 and 900 °C under argon gas(scale bar in the inset of (c) in c is 20 nm).

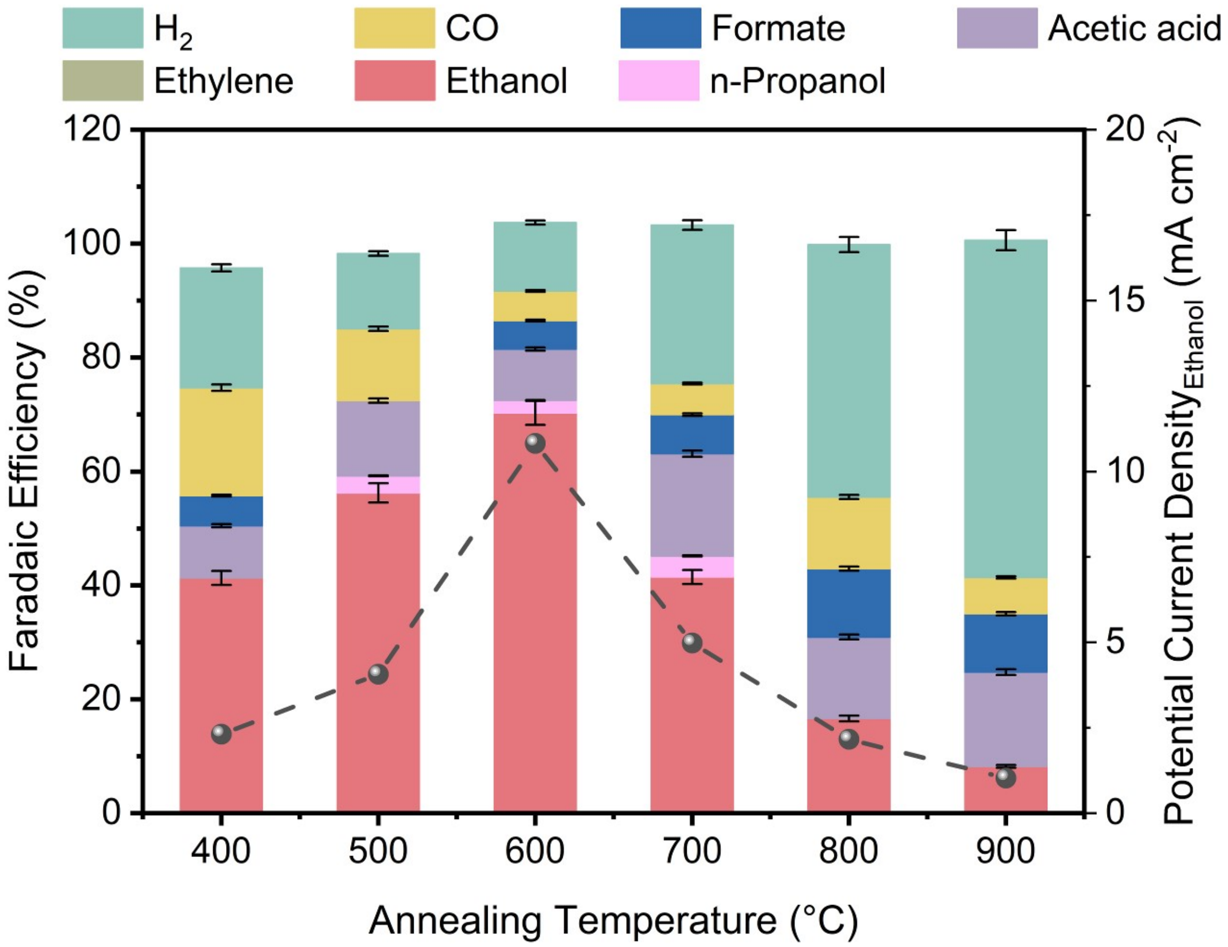

Figure R20. FE and current density over a series of Cu-*x*-N_2 catalysts calcined at different temperatures in nitrogen atmosphere. The experiments were performed at an applied potential of -0.8V vs.RHE in the $KHCO_3$ electrolyte. After one hour of electroreduction, the electrolyte and gas products were collected for analysis. The error bars correspond to the standard deviations of measurements over three separately prepared samples under the same testing conditions.

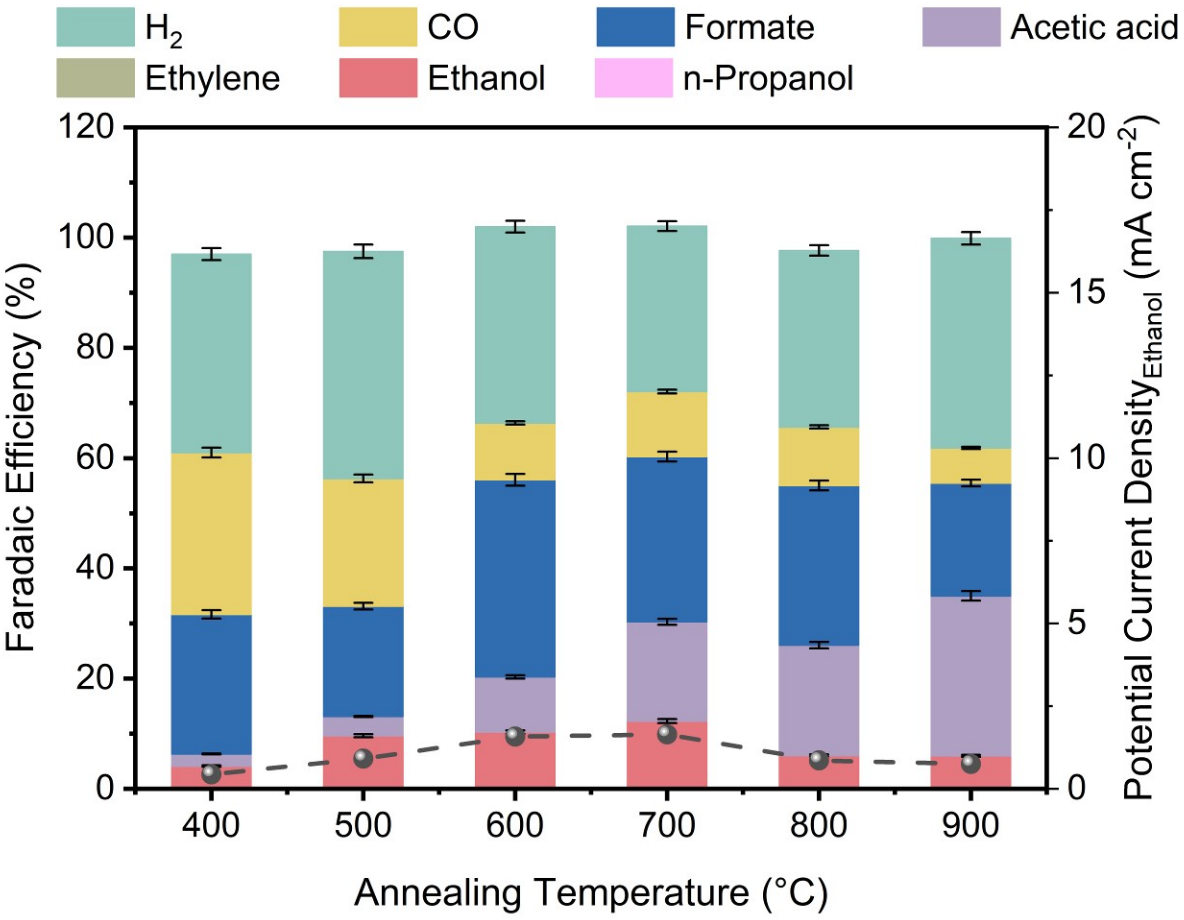

Figure S21. FE and current density at a series of Cu-x-H_2 catalysts calcined at different temperatures in a hydrogen atmosphere. The experiments were performed at an applied potential of -0.8V vs.RHE in the $KHCO_3$ electrolyte. After one hour of electroreduction, the electrolyte and gas products were collected for analysis. The error bars correspond to the standard deviations of measurements over three separately prepared samples under the same testing conditions.

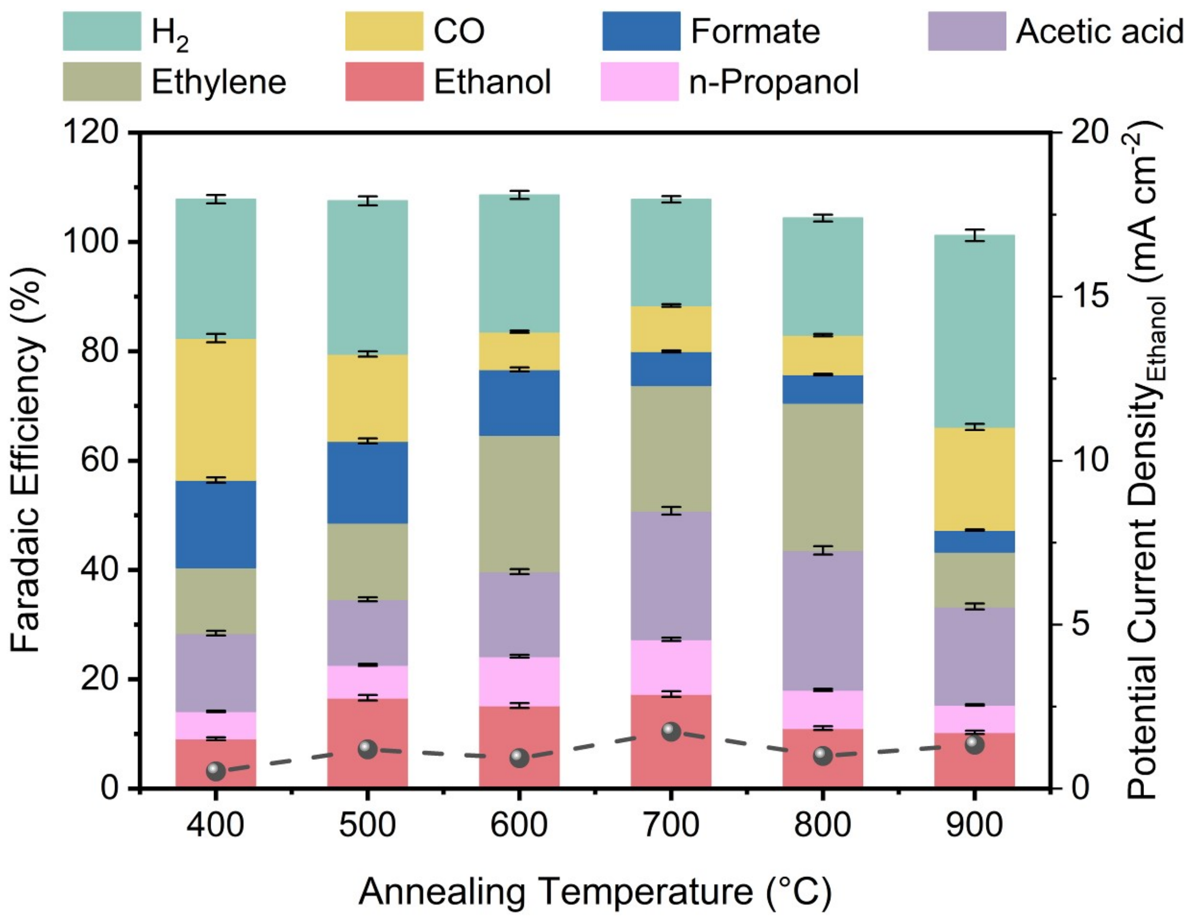

Figure S22. FE and current density at a series of Cu-x-NH_3 catalysts calcined at different temperatures in an ammonia atmosphere. The experiments were performed at an applied potential of -0.8V vs. RHE in the 0.1M $KHCO_3$ electrolyte. After one hour of electroreduction, the electrolyte and gas products were collected for analysis. The error bars correspond to the standard deviations of measurements over three separately prepared samples under the same testing conditions.

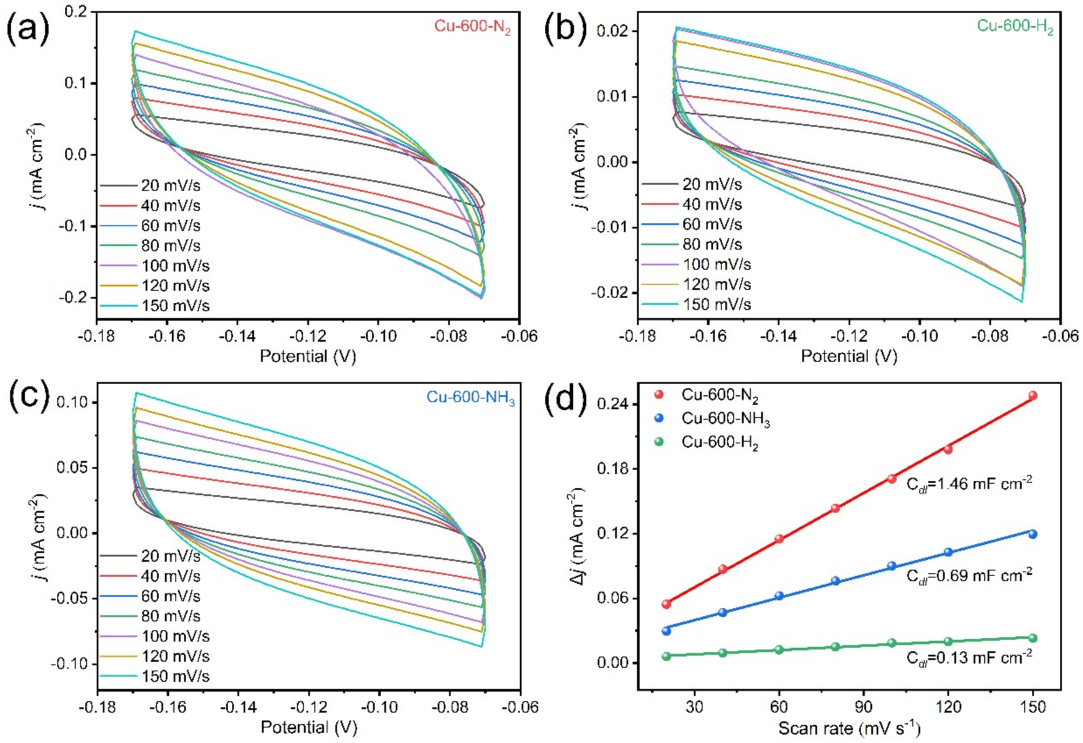

Figure S23. Typical cyclic voltammograms of (a) Cu-600-N_2, (b) Cu-600-H_2, and (c) Cu-600-NH_3 in the non-Faradaic capacitance current range with different scan rates of 20, 40, 60, 80,100,120, and 150 mV s^{-1}. (d) Electrochemically active surface areas are estimated from the double-layer capacitance of the samples. As seen, Cu-600-N_2 exhibits a higher C_{dl} of 1.46 mF cm^{-2} in comparison with other catalysts, suggesting more active sites in Cu-600-N_2.

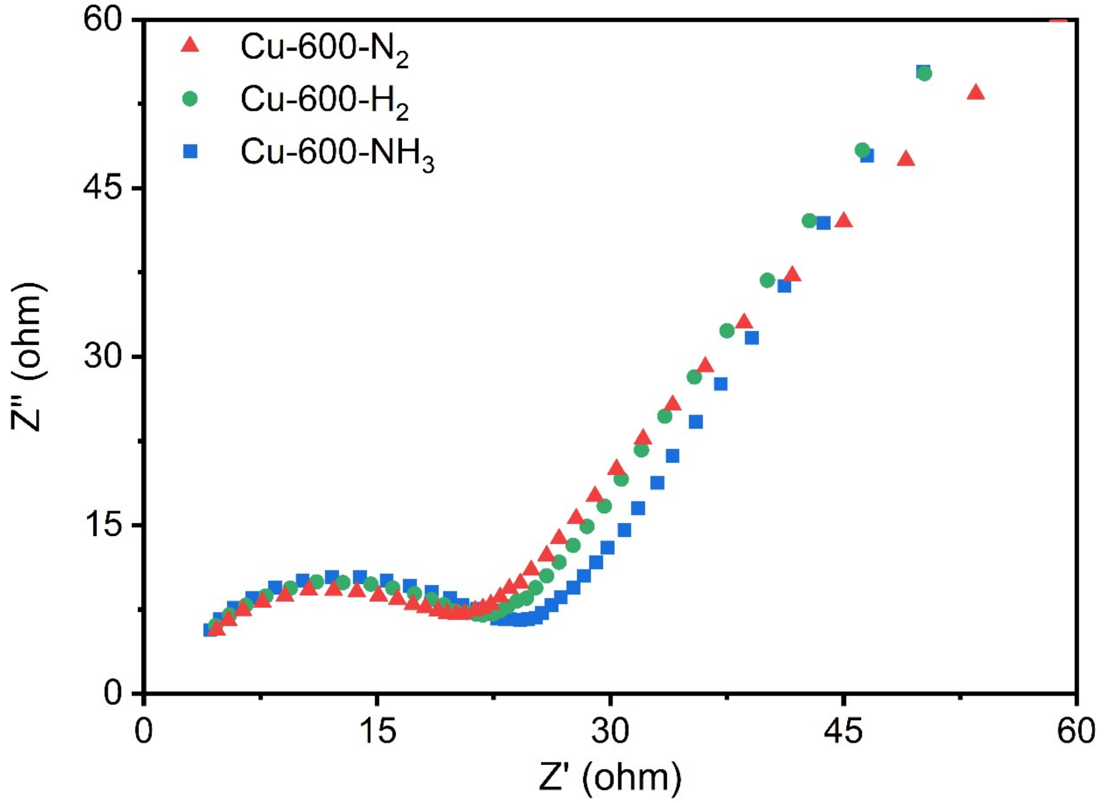

Figure S24. Nyquist plots obtained by electrochemical impedance plots for Cu-600-N_2, Cu-600-H_2, and Cu-600-NH_3. Clearly, it indicates that the charge-transfer resistance of Cu-600-N_2 is lower than that of others, suggesting that Cu-600-N_2 has a faster charge-transfer capacity for reduction.

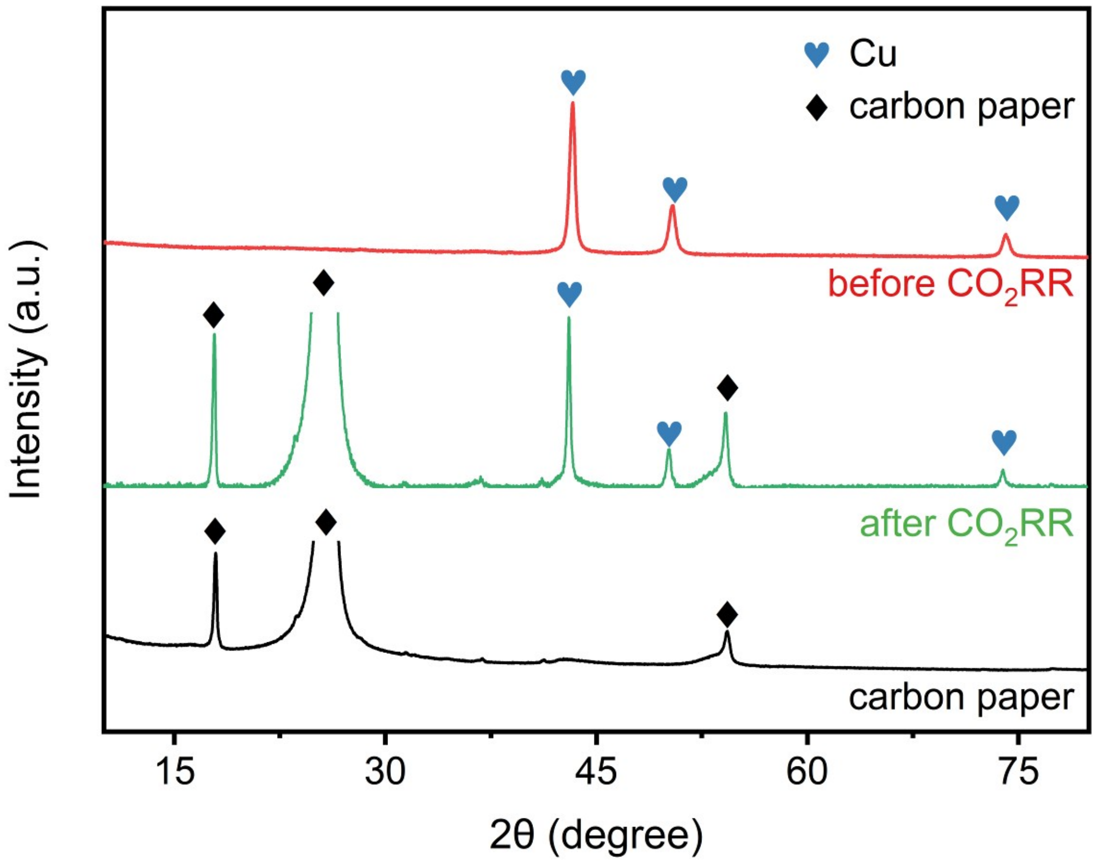

Figure S25. The XRD patterns of catalyst Cu-600-N_2 before and after electrolysis for 3h at -0.8V vs. RHE in 0.1 M $KHCO_3$ electrolyte.

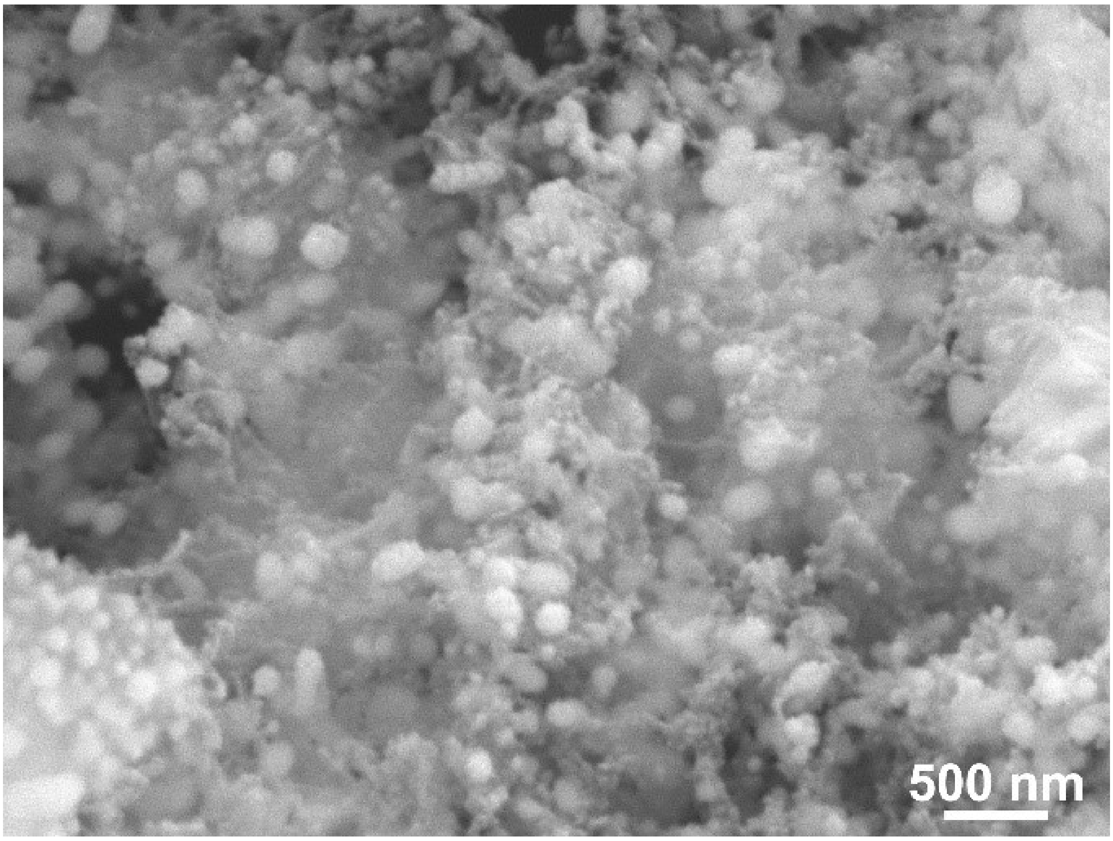

Figure S26. The SEM image of catalyst Cu-600-N_2 after electrolysis for 3h at -0.8V vs. RHE in 0.1 M $KHCO_3$ electrolyte. The structure was stable in the electrochemical reaction.

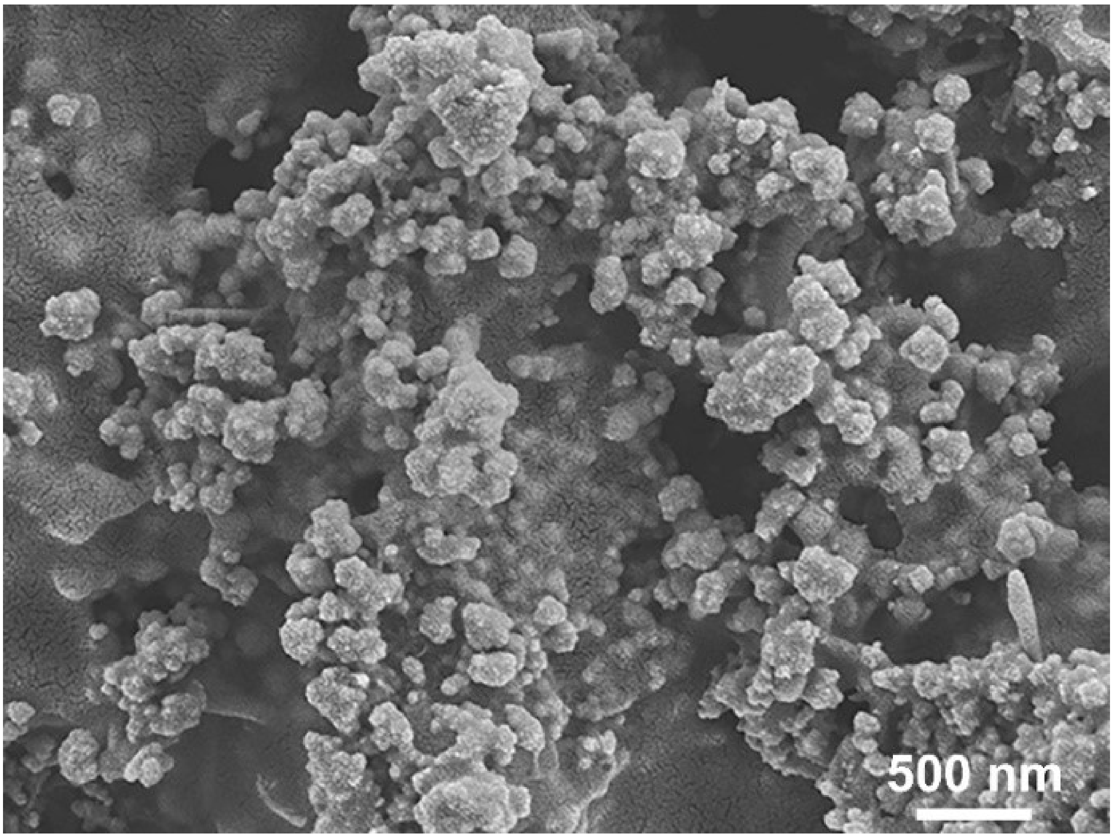

Figure S27. The SEM of catalyst Cu-600-H_2 after electrolysis for 3h at -0.8V vs. RHE in 0.1 M $KHCO_3$ electrolyte. The Cu nanoparticles agglomerated into larger particles on the surface of the carbon paper.

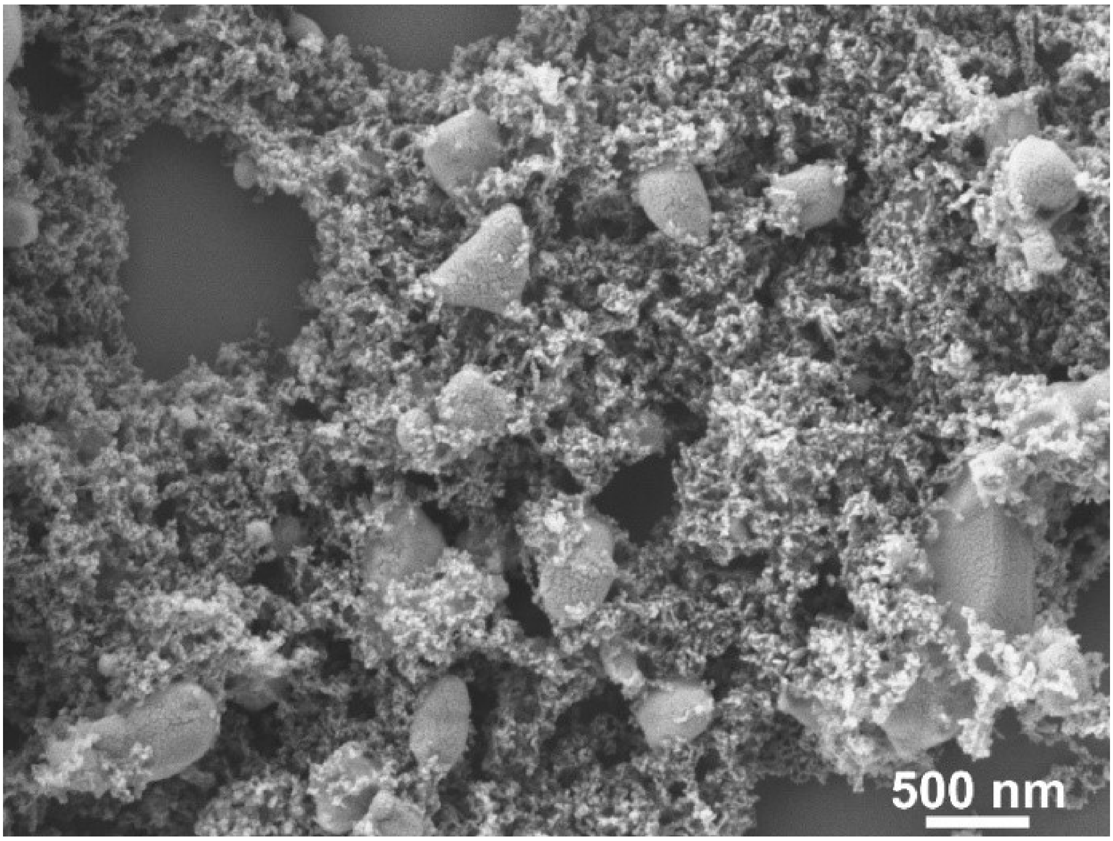

Figure S28. The SEM image of catalyst Cu-600-NH_3 after electrolysis for 3h at -0.8V vs. RHE in 0.1 M $KHCO_3$ electrolyte. The Cu nanoparticles agglomerated obviously.

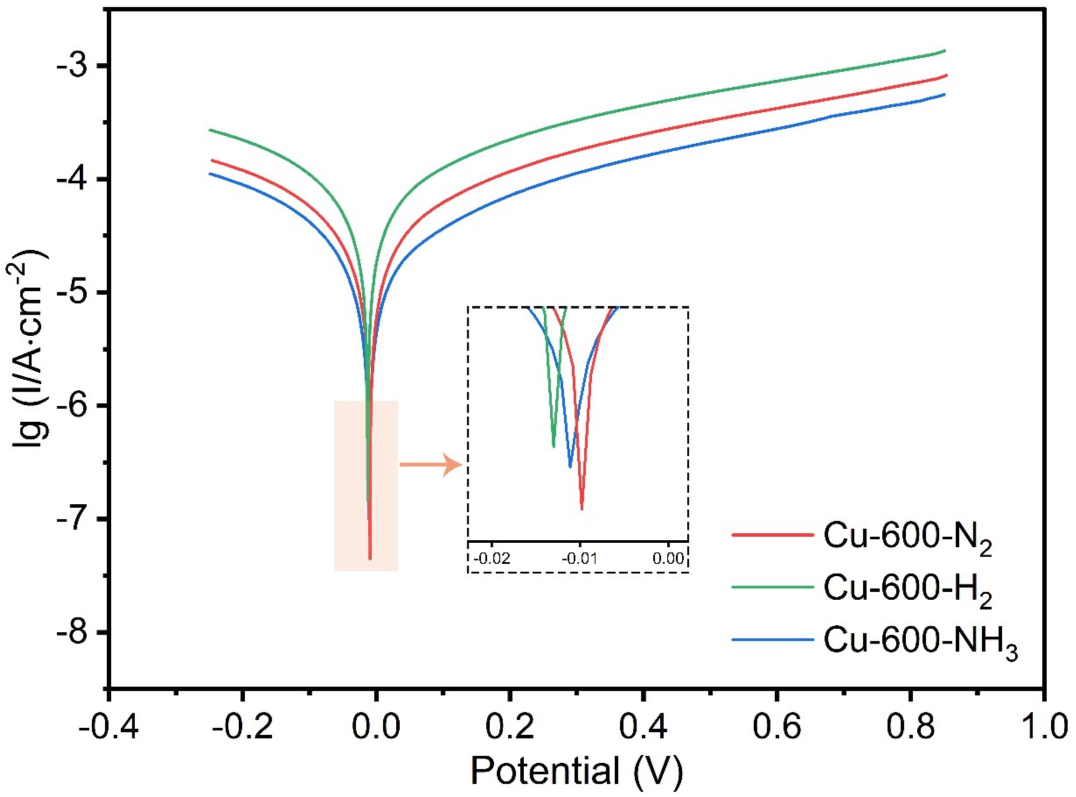

Figure S29. Linear polarization curve of catalysts in an acidic environment. Cu-600-N_2 exhibited the best corrosion resistance among the three catalysts evaluated by Tafel analysis due to its more positive corrosion potential.

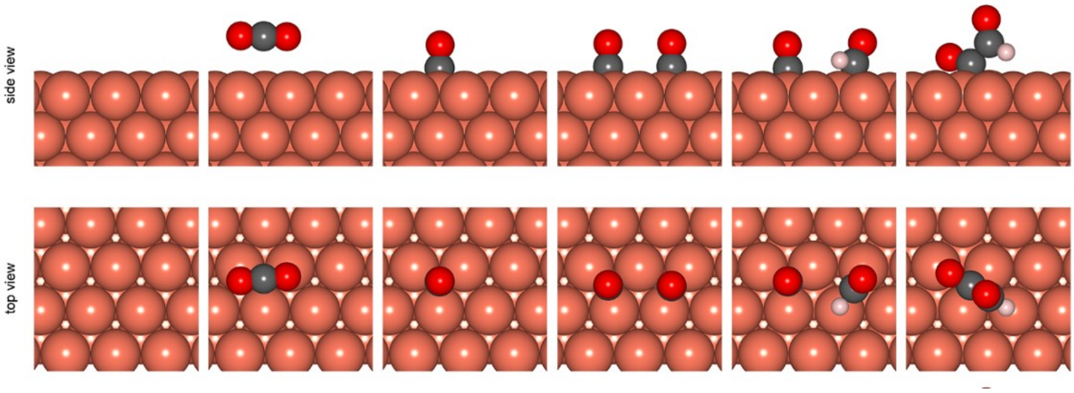

Figure S30. The schematic diagrams of the reaction pathway on a bare Cu surface.

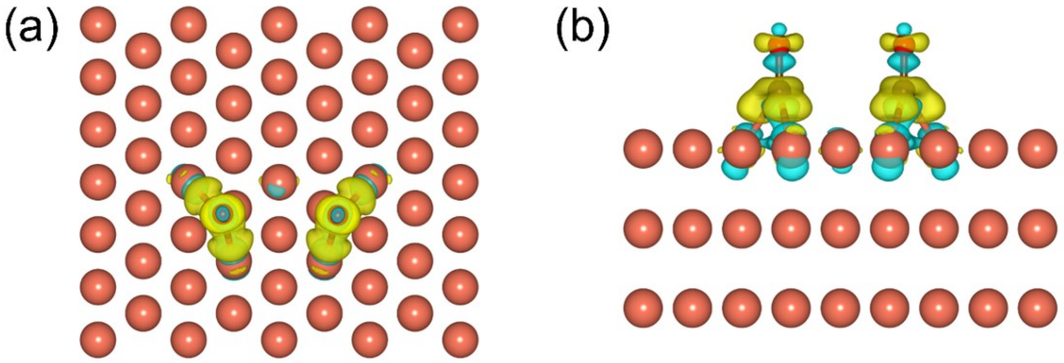

Figure S31. Electron density difference diagrams for Cu with two adsorbed *CO. The top view (a) and side view (b) of charge density difference on the Cu surface.

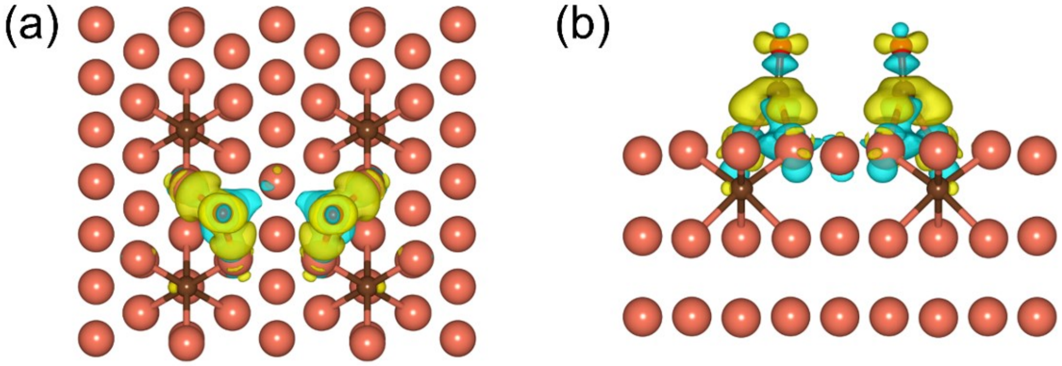

Figure S32. Electron density difference diagrams for Cu-C with two adsorbed *CO. The top view (a) and side view (b) of charge density difference on Cu-C surface. The charge transfer on the Cu-C surface is slightly stronger than on the Cu surface.

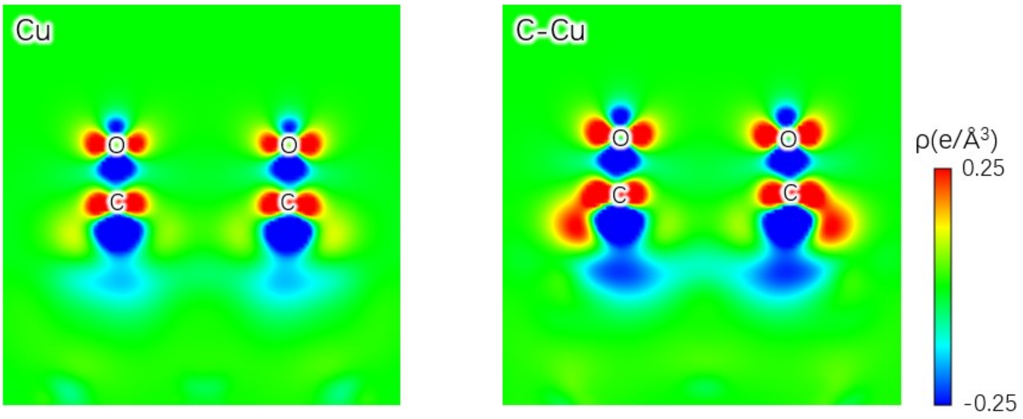

Figure S33. 2D cross-sectional diagrams of the charge density difference for Cu-C and bare Cu, respectively.

Table S1. Contents of Cu in the catalysts were determined by the ICP-OES tests.

Catalyst	Content of Cu (wt%)
Cu-600-N_2	43
Cu-600-H_2	42
Cu-600-NH_3	47

Table S2. Physicochemical properties of the Cu-600-N_2, Cu-600-H_2, Cu-600-NH_3.

Catalyst	S_{BET} (m^2/g)	S_{Micro} (m^2/g)	S_{Micro}/ S_{BET}	V_{Pore} (cm^3 /g)	V_{Micro} (cm^3 /g)	V_{Micro}/V_{Pore}
Cu-600-N_2	1004.63	406.47	40.5%	2.96	0.33	11.1%
Cu-600-H_2	203.25	48.61	23.9%	0.88	0.05	5.6%
Cu-600-NH_3	228.66	120.01	52.5%	0.65	0.09	13.8%

Table S3. Summary of the results derived for the C 1s XPS Spectra of the catalysts

Catalyst	Content (%)				
	C-Cu	sp^2C	sp^3C	C-O	C=O
Cu-600-N_2	49.19	34.39	-	12.78	2.64
Cu-600-H_2	-	76.47	-	14.03	9.49
Cu-600-NH_3	29.89	39.44	13.21	11.46	6.2

Table S4. Comparison of the FE of EtOH and current densities for the Cu-600-N_2 with previously reported Cu-based catalysts in H-type cell.

	Catalyst	j (mA cm^{-2})	FE $_{EtOH}$ (%)	Ref.
	Cu-600-N_2	15.4	67.8	This work
1	CuBr-DDT	12.5	35.9	ACS Energy Lett. 2021, 6, 437−444
2	Cu/CNS	2.1	63	ChemistrySelect 2016, 1, 6055 – 6061
3	GO-VB_6-Cu-2	4.571	56.3	Journal of CO_2 Utilization 33 (2019) 452–460
4	N-ND/Cu	1	28.9	Nat. Nanotech., 2020, 15, 131-137
5	Cu-SA/NPC	6	9	Nat. Commun., 2020, 11, 2455
6	CuZn	0.2	30	Angew. Chem. Int. Ed., 2019, 58, 15036-15040
7	$Cu(OH)_2$/Cu	1	5	ACS Catal., 2019, 9, 6305-6319
8	H-Cu	14	25	ACS Catal., 2019, 9, 5217-5222
9	Cu-Cu_2O	20	55	Nat. Commun., 2019, 10, 3851
10	$Cu_{50}Ag_{50}$	0.75	7	ACS Energy Lett., 2018, 3, 2947-2955
11	CuOx-Vo	15	10	Small Methods., 2018, 1800449
12	Cu-BDD	0.1	42.4	Electro. Acta., 2018, 266, 414-419
13	Rec-Cu	18	15	Adv. Mater., 2018, 1804867
14	Cu NC cubes	7	8	Angew. Chem. Int. Ed., 2016, 55, 5789-5792
